# Rotational cavitator: advances and applications in cavitation-enhanced technologies

**DOI:** 10.1016/j.ultsonch.2025.107727

**Published:** 2025-12-19

**Authors:** Yu-Hang Zhang, Zhi-Ying Zheng, David Ezekoye, Lu Wang, Li-Ming Yao, Vladimir A. Kulagin, Jian Wu

**Affiliations:** a School of Energy Science and Engineering, Harbin Institute of Technology, Harbin 150001, China; b College of Aerospace and Civil Engineering, Harbin Engineering University, Harbin 150001, China; c Institute of Advanced Technology, Heilongjiang Academy of Sciences, Harbin 150020, China; d Department of Heat Technology and Fluid Dynamics, Siberian Federal University, Krasnoyarsk 660041, Russia

**Keywords:** Rotational cavitator, Biofuel, Droplet emulsification, Food processing, Wastewater sludge, Process intensification

## Abstract

Driven by the increasing demand for efficient, energy-saving and sustainable processing technologies, rotational cavitators have shown considerable promise across a range of industrial applications. By inducing intense shear forces and turbulent flow through high-speed rotation in liquid media, rotational cavitation enables strong cavitation effects under controlled conditions, thereby intensifying transport and reaction processes under lower energy input. This review systematically examines the fundamentals, cavitation characteristics and structural evolution of rotational cavitators, with a particular focus on their applications in biofuel production, droplet emulsification, food processing, wastewater treatment and other process intensification. The review comprehensively discusses the benefits of rotational cavitation in multiple application domains, outlines current research progress and emerging trends, and provides theoretical insights and practical guidance for future research and industrial implementation.

## Graphical abstract

**Figure:**
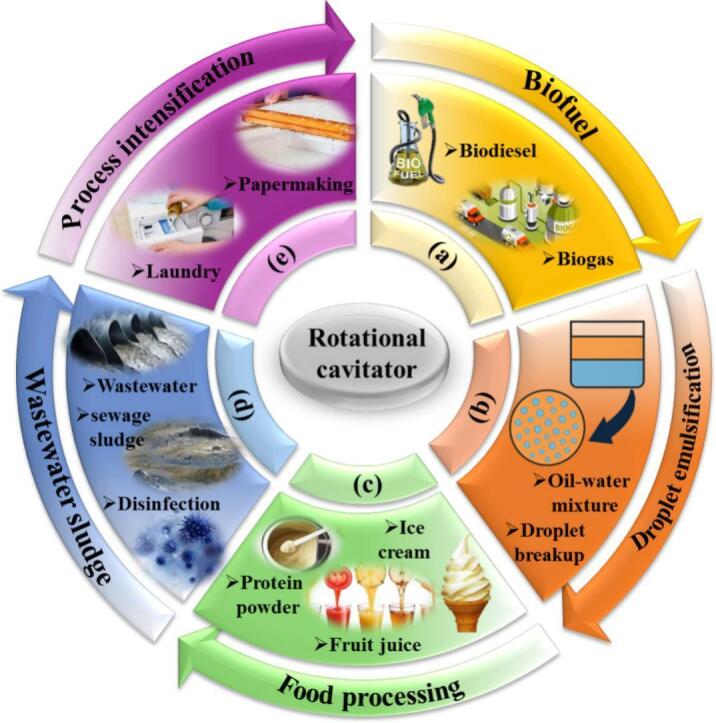

## 1 Introduction

With the rapid advancement of industrial manufacturing, the scale and complexity of resource processing have increased dramatically, often accompanied by environmental pollution, resource loss, and low energy efficiency [[1], [2], [3], [4], [5]]. These challenges have intensified the need for efficient, energy-saving, and sustainable technologies [6,7]. Cavitation, characterized by high energy density and localized effects, has emerged as a promising non-conventional approach [[8], [9], [10]]. It involves the nucleation, growth, and collapse of bubbles triggered by pressure fluctuations, generating localized high temperature, high pressure, shear, and free radicals that can enhance heat/mass transfer and disrupt material structures [[11], [12], [13], [14], [15]].

Rotational cavitators, which induce transient low-pressure zones via high-speed rotor–stator systems, have gained increasing attention due to flexible control, tunable intensity, and relatively low energy demand [16,17]. Compared with conventional hydrodynamic and ultrasonic devices, they form stable cavitation zones at lower input, avoid side reactions, and deliver higher efficiency [[18], [19], [20], [21]]. These advantages have driven their transition from laboratory studies to engineering applications across wastewater treatment [22,23], biofuel production [24], emulsification [25], and food processing [26], enabling cleaner and more sustainable solutions [[27], [28], [29]].

To clarify its mechanistic features and support deeper insight, this review first outlines the principles of cavitation and the operational characteristics of rotational cavitators. It then provides a comprehensive overview and critical analysis of recent progress in five key areas: biofuel production, droplet emulsification, food engineering, wastewater treatment, and other process intensification fields. The objective is to uncover the fundamental mechanisms and performance advantages of rotational cavitators, offering theoretical foundations and technological perspectives to inspire interdisciplinary innovation.

## 2 Hydrodynamic cavitation mechanisms

### 2.1 Cavitation effects

Cavitation, first described by Euler in 1754 and later linked to propeller erosion by Reynolds and Parsons, has since become a key engineering phenomenon [[30], [31], [32], [33], [34]]. It is now recognized as a four-stage process—nucleation, growth, migration, and collapse [[35], [36], [37]]—where bubble implosion releases concentrated energy, producing physical (shock waves up to 500 MPa, microjets >100 m/s, water hammer >400 MPa, shear ∼3.5 kPa [19,[38], [39], [40]]), thermal (hotspots of several-thousand K; heating rates up to 10^10^ K/s [32,41]), and chemical effects (water dissociation and ^•^OH radical formation [[42], [43], [44]]). Collectively, these extreme microscale conditions enable hydrodynamic cavitation (HC) to enhance mass/heat transfer, disrupt materials, and accelerate reactions. Compared with conventional methods, HC offers low energy demand, no added chemicals, strong intensification, and compact, tunable equipment, underpinning its potential in environmental, food, and bioprocessing sectors [[45], [46], [47], [48], [49]].

### 2.2 Rotational cavitator

Early studies treated cavitation mainly as an engineering failure, but it has since evolved into a tool for environmental remediation and process intensification [[50], [51], [52]]. To achieve controllable cavitation, two main hydrodynamic designs have been developed: non-rotational (venturi, orifice) and rotational (rotor–stator or rotor–rotor) cavitators [16,37,[53], [54], [55]]. While venturi/orifice types are simple, they suffer from weak cavitation, low throughput, and scaling challenges [12,23,56]. In contrast, rotational cavitators use high-speed rotors to generate strong shear and turbulence, creating intense and tunable cavitation under continuous flow [57,58]. Their cavitation zone lies in the rotor–stator shear gap, where rapid pressure drops induce bubble formation and violent collapse [25,59]. Depending on geometry, they can be radial or axial, with common designs including indentation, serrated, pinned-disk, and hybrid structures [22]. Compared with conventional devices, rotational cavitators provide higher mass transfer, lower energy demand, stronger cavitation intensity, and greater flexibility, enhancing applications in material disruption [60], chemical reactions [56], emulsification [61], and pollutant degradation [62].

### 2.3 Dimensionless parameters for cavitation assessment

#### 2.3.1 Cavitation number

The cavitation number is a key dimensionless parameter that characterizes the balance between the pressure field and the flow velocity, and is widely used to determine the onset and intensity of cavitation. In rotational cavitators, cavitation formation is jointly governed by rotor speed, shear rate, vortex-induced local low-pressure zones, and the overall flow distribution. In general, a lower cavitation number (i.e., local pressure approaching or falling below the vapor pressure) corresponds to a higher propensity for cavitation inception and development. The cavitation number is defined as:

(1) $$C_{v}=\frac{p-p_{v}}{\left(1/2\right)\rho v^{2}}$$

where *p* and *v* are the absolute pressure and velocity at a selected location, $p_{v}$ is the saturated vapor pressure of the working fluid, and ρ is the liquid density.

It should be noted that the internal flow in rotational cavitators is non-uniform and cavitation is governed by multiple coupled factors. Consequently, different studies adopt different choices for *p* and *v* depending on device geometry, measurement location, and modelling objectives. For example, some authors use the tangential velocity at the rotor face, others use the inlet velocity, while some take the local velocity in the rotor–stator gap as the characteristic velocity. The pressure term may be taken as the inlet pressure, outlet pressure, or a local pressure within the shear zone. As a result, various forms of cavitation number definitions have been proposed in the literature. For clarity and cross-comparison, the most commonly used definitions of cavitation number in rotational cavitation studies are summarized in Table 1, covering different strategies for selecting the pressure and velocity terms, and providing a reference for future work.

**Table 1:** Various definitions of the cavitation number.

Cavitation number definition	Description of parameters	Ref.
$C_{v}=\frac{p_{0}-p_{v}}{\left(1/2\right)\rho v_{r}^{2}}$	$p_{0}$: pressure at the indentation inlet $v_{r}$: tangential velocity on the rotor surface	[63]
$C_{v}=\frac{p_{2}-p_{v}}{\left(1/2\right)\rho v^{2}}$	$p_{2}$: downstream pressure at the reactor outlet v: surface flow velocity on the rotor surface	[64]
$C_{v}=\frac{p_{2}-p_{v}}{\left(1/2\right)\rho v_{\mathrm{ind}}^{2}}$	$p_{2}$: absolute inlet pressure $v_{\mathrm{ind}}$: fluid velocity at the point of cavitation	[65]
$C_{v}=\frac{p_{\mathrm{out}}-p_{v}}{\left(1/2\right)\rho {\left(v_{\mathrm{in}}+v_{\mathrm{tangential}}\right)}^{2}}$	$p_{\mathrm{out}}$: static pressure at the outlet $v_{\mathrm{in}}$: inlet velocity $v_{\mathrm{tangential}}$: rotor tangential velocity	[66]
$C_{v}=\frac{p_{1}-p_{v}}{\rho v^{2}}\times 500$	$p_{1}$: inlet pressure v: inlet velocity	[67]
$C_{v}=\frac{p_{\infty }-p_{v}}{\left(1/2\right)\rho v_{\infty }^{2}}$	$p_{\infty }$: static pressure in the undisturbed reference section $v_{\infty }$: fluid velocity in the undisturbed reference section	[68]

#### 2.3.2 Rotational Reynolds number

The rotational Reynolds number is an important dimensionless parameter in rotational cavitation systems. It quantitatively evaluates the relative magnitude of rotation-induced inertial forces to viscous damping, thereby indicating the extent to which rotational motion influences the internal flow structure and cavitation behavior. It is generally defined as:

(2) $${\text{Re}}_{\Omega }=\frac{(R\omega )\;l}{\nu }$$

where ω is the angular velocity of the rotor, *R* is the rotor radius, *l* is a characteristic length scale, and ν is the kinematic viscosity of the fluid.

In rotational cavitators, the specific calculation of the rotational Reynolds number depends on the device geometry and operating conditions. For clarity and comparison, commonly used definitions and parameter-selection strategies are summarized in Table 2.

**Table 2:** Various definitions of the rotational Reynolds number.

Rotational Reynolds number definition	Description of parameters	Ref.
${\text{Re}}_{\Omega }=\frac{\left(R_{1}\omega \right)\left(R_{2}-R_{1}\right)}{\nu }$	$R_{2}$: inner radius of the stator $R_{1}$: outer radius of the rotor (e.g., at the rotor–indentation edge)	[63]
${\text{Re}}_{\Omega }=\frac{R_{2}^{2}\omega }{\nu }$	$R_{2}$: outer radius of the system	[69]
${\text{Re}}_{\Omega }=\frac{NH^{2}}{4\nu }$	*N*: rotor rotational speed *H*: gap width between the rotor and stator	[70]

## 3 Biofuel

**Fig. 1:**
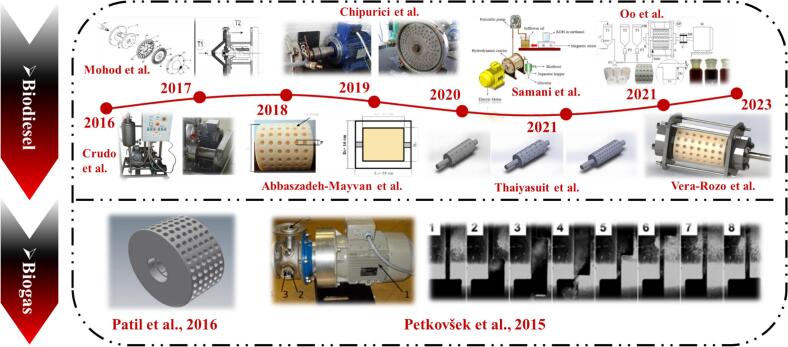
Types of rotational cavitators applied in biofuel production [59,61,74,[77], [78], [79], [80], [81], [82], [83]].

With rising crude oil prices, depletion of fossil resources, and worsening environmental pressures, the need for alternative energy is urgent [71,72]. Biofuels are attractive due to renewability, low emissions, and biodegradability [73]. As a process-intensification device, the rotational cavitator boosts biodiesel synthesis and biogas fermentation (Fig. 1). In biodiesel production, cavitation-induced microscale mixing accelerates transesterification, enabling high conversions without external catalysts or harsh conditions [74,75]. In biogas systems, cavitation generates strong shear and micro-jets that disrupt cells and, via water splitting, forms reactive radicals that promote biomass degradation [76,77]. This green pathway simplifies processing and lowers operating costs, supporting a sustainable bioenergy framework.

### 3.1 Biodiesel

Essentially, biodiesel consists of fatty acid ester compounds formed by transesterifying animal/vegetable oils with alcohols (e.g., methanol or ethanol) in the presence of a catalyst; key levers are the alcohol-to-oil molar ratio, catalyst type, and loading [84]. Conventional stirred processes require long times, excess reactant, and high energy, limiting competitiveness. Current efforts focus on two fronts [80]: (1) lowering feedstock cost by using waste cooking oil to valorize waste lipids and cut raw-material expense [85,86]; and (2) applying process-intensification to reduce reagent use and energy. Among these, rotational hydrodynamic cavitation provides efficient mixing and micromixing with good scalability, enabling shorter residence times and higher yields at lower energy input—offering a practical route toward industrial biodiesel production [87,88].

**Fig. 2:**
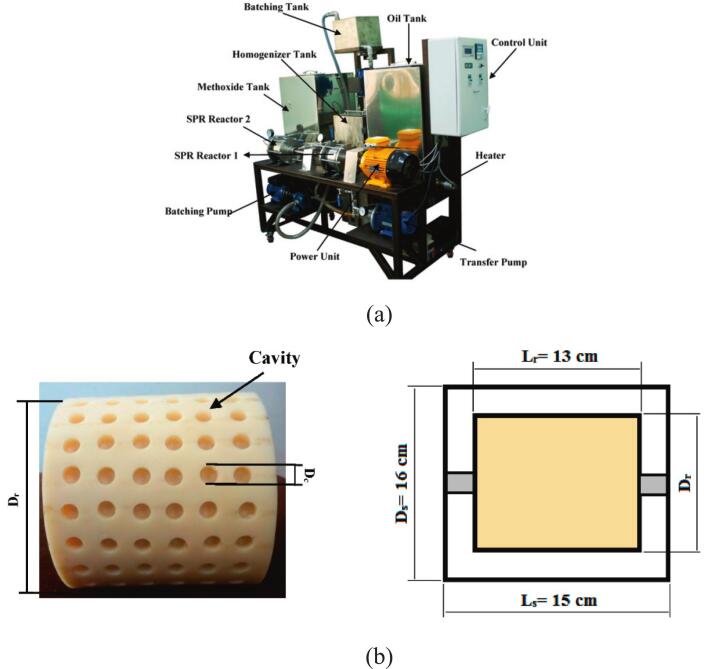
(a) Rotational cavitation device; (b) Rotor structure diagram [80].

To improve the efficiency and reduce the cost of biodiesel production, Crudo et al. [78] developed a rotational cavitator that generates micron-scale liquid droplets, significantly enhancing mass and heat transfer and enabling high-purity biodiesel conversion. Compared with conventional orifice reactors, the system eliminates clogging risks, achieves a circulation rate of 390 L/h, and requires only 10 s per cycle. The specific energy consumption is 0.030 kWh/L, an 86 % reduction relative to 0.222 kWh/L, with favorable scale-up potential and spatial adaptability. Abbaszadeh-Mayvan et al. [80] further proposed a series-connected rotational cavitator process for continuous waste cooking oil (WCO)-to-biodiesel production, with operating variables optimized by response surface methodology (RSM). The core rotor–stator design features uniformly distributed indentations on the rotor (Fig. 2). During high-speed rotation, liquid periodically enters these cavities and experiences abrupt pressure fluctuations, initiating cavitation inception and collapse. The resulting intense mixing and in-situ energy release improve interfacial mass transfer without external heating. Compared with a stirred-tank reactor, conversion is achieved within 30 s, and, relative to ultrasound, the system offers better controllability and is more amenable to continuous and large-scale operation.

**Fig. 3:**
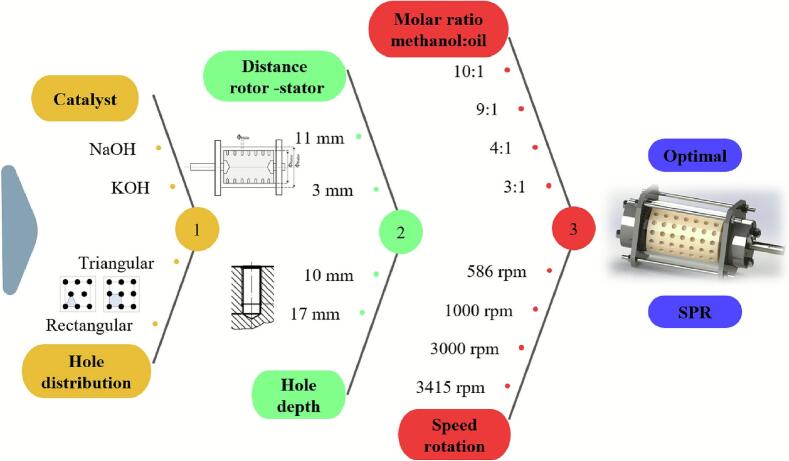
Workflow for optimizing cavitator geometry and reaction parameters [82].

Samani et al. [59] employed an indentation-type rotational cavitator to produce biodiesel from safflower seed oil and examined the effects of reaction time, KOH loading, alcohol-to-oil molar ratio, and rotor–stator gap on yield. Under optimal conditions, the yield reached 89.11 %, meeting international specifications, and outperforming an ultrasonic reactor in both yield and efficiency. Similarly, Thaiyasuit et al. [81] produced fatty acid methyl esters (FAME) continuously from waste cooking oil at room temperature; at area fractions (AF) = 27.6 % (120-indentation rotor), 3000 rpm, 1.5 % w/w base catalyst, and 2.027 L/min flow, the FAME yield was 98.6 % with a specific energy of 12.5 W·h/kg. Energy use was lower than that of an orifice hydrodynamic cavitator, ultrasonic reactor, and stirred tank by 93.2 %, 95.0 %, and 97.5 %, respectively, and the product met EN 14214 (the European quality standard for FAME biodiesel) and ASTM D6751 (the U.S. specification governing the quality requirements for B100 biodiesel fuel) standards. Likewise, Vera-Rozo et al. [82] optimized cavitator geometry and operating parameters (Fig. 3); a rectangular indentation pattern, 17 mm indentation depth, 3 mm rotor–stator gap, and NaOH catalyst formed the best combination, enabling an indentation-type rotational cavitator to achieve in 6 min the conversion typically obtained in 120 min by conventional processing.

Mohod et al. [79] used a radial serrated rotational cavitator to intensify biodiesel production and evaluated the effects of operating variables while comparing waste cooking oil (WCO) with fresh sunflower oil (FCO). Under optimized conditions—120 min reaction time, 12:1 methanol-to-oil molar ratio, 3 wt% KOH, and 50 °C—the maximum yields reached 97 % for WCO and 92.3 % for FCO. Chipurici et al. [74] employed a commercial rotational cavitator (SME SC Progen IMPEX SRL) for FAME synthesis using market sunflower oil and Sigma-Aldrich methanol. The rotor disk incorporated uniformly distributed cylindrical pins along its rim, which enhanced mixing and interfacial mass transfer; the combined high shear and strong turbulence enlarged the oil–alcohol contact area and accelerated transesterification.

**Fig. 4:**
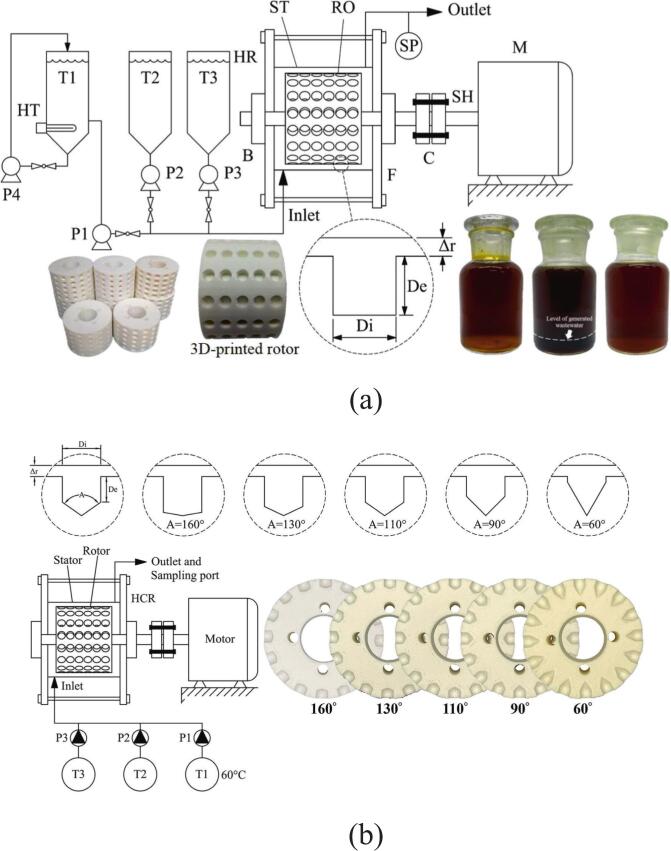
(a) Schematic of the early hydrodynamic cavitation experimental setup [64]; (b) Schematic of the optimized conical rotor design [56].

In biodiesel production, free fatty acids (FFAs) in feedstock oils tend to react with alkaline catalysts, forming soap and thereby reducing transesterification efficiency. To address this, free fatty acid (FFA) levels must be reduced through pretreatment before base-catalyzed reactions. Oo et al. [64] designed a low-cost 3D-printed rotor with spherical indentations (Fig. 4) for a rotational cavitator and, using response-surface optimization, reduced FFA from 11.456 wt% to 1.028 wt%, achieving an esterified-oil yield of 96.07 vol% at a specific energy of 0.0264 kWh/L. Building on this work, Pongraktham and Somnuk [89] implemented a two-step optimization with a 3D-printed plastic rotor: FFA decreased from 89.16 wt% to ≈36.69 wt% in step one (60.8 % methanol, 7.2 % H_2_SO_4_, 5.0 mm indentation diameter, 6.1 mm indentation depth, 3000 rpm) and then to 0.94 wt% in step two (44.5 % methanol, 3.0 % H_2_SO_4_, 4.6 mm indentation diameter, 5.8 mm indentation depth, 3000 rpm). The combined process delivered >93 % esterified-oil yield, an average specific energy of 0.137 kWh/L, and a total reaction time of 61 s. To further enhance pretreatment, Oo and Somnuk [56] replaced spherical indentations with apex-cone (conical) indentations and analyzed the effects of indentation diameter, depth, and cone angle on FFA removal (Fig. 4). Under optimized conditions, FFA decreased from 12.014 wt% to ≈1 wt%, with 97.34 vol% esterified-oil yield and 93.31 vol% purified-oil yield. The redesign also reduced reagent costs by $0.94 h^−1^ for methanol and $0.10 h^−1^ for sulfuric acid. Overall, conical-indentation cavitators outperformed cylindrical- indentation designs and clarified the link between cavitator geometry and pretreatment performance, offering a high-efficiency, low-cost, and scalable green route for biodiesel feedstock conditioning (see Table 3 for further details).

**Table 3:** Applications of rotational cavitators in biodiesel production.

Feedstock	Parameters	Performance	Mechanism	Ref.
Refined/ decolorized palm oil, WCO	55 °C; NaOH 3.67 g/L oil; 390 L/h; two-step methanol (75 % + 25 %); circulation: 15 + 15 min	Micron droplets; FAME >99 %; lower energy use	Bubble collapse creates micron dispersion, enhancing mixing/transfer; faster transesterification	[78]
WCO	*Dr*/*Ds* = 0.73, *Dc*/*Dr* = 0.06, *dc*/Δ*r* = 0.50; 25510.55 rpm; 30.10 s	FAME: 98.53 %	Local low-pressure generation and collapse accelerate reaction; cavitation shortens residence	[80]
Safflower oil	63.88 s; KOH 0.94 wt%; methanol-to-oil molar ratio: 8.36:1; gap: 1.53 cm	Biodiesel: 89.11 %; met EN 14214 and ASTM D6751 standards	Cavitation increases interfacial area and accelerates esterification	[59]
WCO	3000 rpm; catalyst: 1.5 % w/w; methanol-to-oil: 6:1; 2.027 L/min	FAME: 98.6 %; energy consumption: 12.5 W·h/kg	Indentation rotor boosts turbulence/cavitation; collapse intensifies mixing/transfer	[81]
Soybean oil	Rectangular indentation pattern; depth: 17 mm; gap: 3 mm; NaOH; 2000–3415 rpm; methanol/oil: 6.5:1–10:1; 6 min	FAME: 97.63 %; shorter time; lower energy	Cavitation generates shockwaves to intensify mixing and promotes transesterification	[82]
Waste/new edible oils	Methanol/oil: 12:1; KOH: 3 wt%; 50 °C; 120 min	Yield: 97 % (WCO); 92.3 % (fresh)	High shear and cavitation promote immiscible mixing and transesterification	[79]
Sunflower oil	Alcohol/oil: 6:1; NaOH: 0.25–1 wt%; 40 °C; residence:50–180 s	Higher FAME	cavitation enhances mixing and heat transfer, improving transesterification	[74]
Mixed palm oil with high FFA	Methanol: 17.7 vol%; H_2_SO_4_: 2.9 vol%; 3000 rpm; indentation diameter: 4 mm; depth: 6 mm	FFA reduced from 11.46 % to 1.03 %; esterified-oil: 96.07 %	Shear/turbulence at indentations create low-pressure zones; cavitation promotes esterification	[64]
Sludge palm oil (FFA 89.16 %)	Step 1: methanol 60.8 vol%, H_2_SO_4_ 7.2 vol%, indentation diameter 5 mm, depth 6.1 mm, 3000 rpm; Step 2: methanol 44.5 vol%, H_2_SO_4_ 3.0 vol%, indentation diameter 4.6 mm, depth 5.8 mm, 3000 rpm	FFA reduced to 36.69 wt% (Step 1), then to 0.94 wt% (Step 2); 0.137 kWh/L	Two-step cavitation enhances micro-mixing/contact, enabling staged FFA removal	[89]
Mixed crude palm oil (FFA 12.01 %)	Methanol: 20.8 wt%; H_2_SO_4_: 2.6 wt%; 3000 rpm; indentation diameter: 5 mm; depth: 5 mm; cone angle: 110°	FFA reduced to ∼1 wt%; esterified-oil: 97.34 vol%; pure-oil: 93.31 vol%	Cavitation induced by conical indentations generates localized high pressure and temperature, enhancing esterification and mixing efficiency	[56]

### 3.2 Biogas

**Fig. 5:**
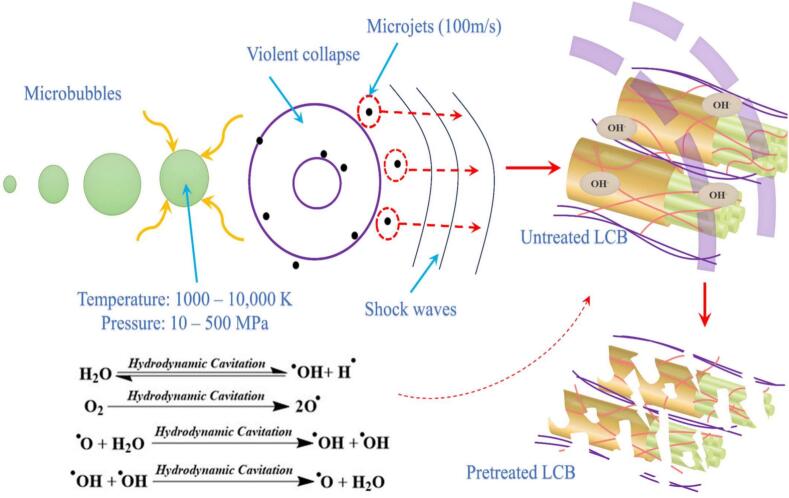
Effects of cavitation on the degradation of lignocellulosic structure [27].

Similar to biodiesel, biogas is a renewable energy source with strong potential to replace conventional fossil fuels, especially in regions rich in agricultural resources. It can be efficiently produced from lignocellulosic biomass such as crop residues, offering significant environmental and energy benefits [90,91]. Currently, anaerobic digestion remains the primary industrial pathway for biogas production from organic waste [92,93]. However, lignocellulosic materials like wheat straw exhibit strong resistance to degradation due to their complex and compact structure, which limits hydrolysis rates and overall gas yield [94,95]. To improve biodegradability and biogas output, pretreatment is often required to break down structural barriers and enhance microbial access to organic matter [96]. Therefore, developing efficient, low-energy, and industrially viable pretreatment methods is critical. Cavitation treatment represents a feasible approach, as illustrated in Fig. 5. The intense forces and oxidative radicals generated by cavitation bubbles can effectively disrupt the structural barriers of lignocellulose, thereby facilitating subsequent processing steps.

Beyond acting as standalone intensification tools, rotational cavitators also work synergistically with other methods. Using an indentation-type cavitator, Patil et al. [77] optimized speed, solids loading, and residence time for biogas pretreatment. Straw concentrations above 1.5 % caused clogging; the optimal cavitation intensity occurred at 2300–2500 rpm. Methane yield increased from 31.8 mL to 77.9 mL after cavitation, and rose to more than fivefold of the baseline when combined with alkaline treatment. The process generates hydroxyl and peroxyl radicals, which aid lignin degradation. Based on the same mechanism, Garuti et al. [97] deployed a commercial cavitator (Three-ES, Italy) at full scale in an agricultural biogas plant. Hydrodynamic cavitation increased methane production by ≈10 %, improved digestate rheology, and lowered mixing, heating, and pumping energy; stable methane output was achieved at a low specific energy of 470 kJ/kgTS. In a follow-up full-scale comparison, Garuti et al. [98] further conducted a systematic assessment of four mechanical pretreatment methods—knife milling, hammer milling, extrusion, and shearing combined with hydrodynamic cavitation—in an industrial biogas plant, examining their effects on feedstock physical properties, energy consumption, and anaerobic digestion performance. The results showed that knife milling achieved the most pronounced particle-size reduction, followed by hydrodynamic cavitation, while the latter was not suitable for high-solids substrates. Nevertheless, when evaluated holistically, hydrodynamic cavitation was confirmed to be effective and practically valuable for enhancing biogas yield and improving overall energy efficiency. Petkovšek et al. [83] treated waste activated sludge (WAS) with a counter-rotating radial-serrated cavitator. After 20 treatment cycles, the soluble chemical oxygen demand (SCOD) increased from 45 mg/L to 602 mg/L, and biogas production rose by 12.7 %. Cavitation promoted soluble organics release and supplied more readily degradable substrates for anaerobic digestion (see Table 4 for further details).

**Table 4:** Applications of rotational cavitators in biogas production.

Feedstock	Parameters	Performance	Mechanism	Ref.
Wheat straw	2300–2700 rpm; treatment 2–6 min; straw-to-water: 0.5 %–1.5 %	Biogas increased by 144 %	Rotor-induced cavitation disrupts lignocellulose, promotes cellulose/hemicellulose hydrolysis and lignin oxidation	[77]
Agricultural residues (e.g., corn)	input: 470 kJ/kgTS; operation: ∼6 months	Methane increased by ∼10 %; digestate viscosity and particle size reduced	Cavitation shear and turbulence enhance lignocellulose breakdown and biodegradability	[97]
Corn, sorghum, cattle manure, /Slurry	Energy consumption: 28.8 kJ/kg	Methane increased by 4 %; particles (>5 mm) decreased by 50 %; surface increased by 9 %	Bubble collapse disrupts cellulose crystallinity, increases porosity, accelerates hydrolysis	[98]
WAS	2850 rpm; gap: 0.8 mm; 20 cycles	SCOD: 45 mg/L to 602 mg/L; soluble Kjeldahl nitrogen: 6.3 mg/L to 71 mg/L; biogas increased by 12.7 %	Cavitation collapse lyses cells, releases organics, enhances solubilization and anaerobic digestion	[83]

## 4 Droplet emulsification

**Fig. 6:**
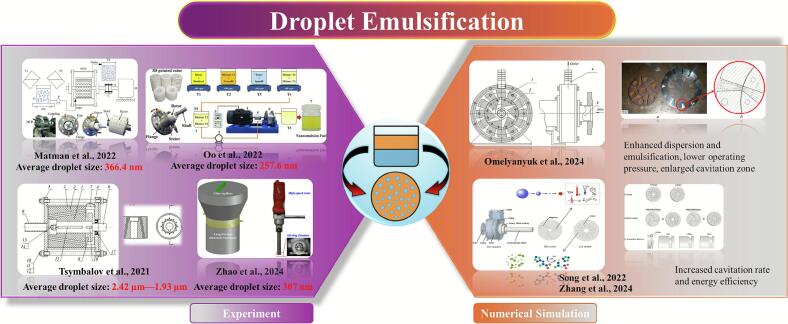
Research progress on the application of rotational cavitators in droplet emulsification [25,61,[104], [105], [106], [107], [108]].

Droplet emulsification, essential in sectors from cosmetics to materials synthesis, converts immiscible liquids (typically oil and water) into stable oil-in-water (O/W) or water-in-oil (W/O) systems [29,[99], [100], [101]]. Conventional methods (stirring, high-pressure homogenization) allow only partial droplet control and involve high energy use with limited scalability [102,103]. Rotational cavitators provide an efficient alternative: intense shear and pressure fluctuations disrupt oil–water interfaces, enabling nano- or submicron emulsions with improved stability and reduced energy demand [25,61]. These advantages make them a competitive technology for advanced emulsification (Fig. 6).

Early investigations by Santos et al. [109] assessed the performance of a novel rotational cavitator designed by their team for producing nanoemulsions from industrial-grade liquid paraffin. The reactor featured a rotor–stator assembly, with both components constructed as grooved disks uniformly patterned along the circumference. It produced oil–water emulsions with an average droplet size of ∼590 nm, stability beyond one month, and a throughput of ∼1.02 m^3^/h, at low energy input. Subsequently, Rinaldi et al. [110] applied a rotational cavitator to waste-cooking-oil reuse. Air injection during hydrodynamic cavitation promoted oxidative polymerization and emulsification, yielding fatliquor precursors for the leather industry. Stable emulsions formed without surfactant, and oxidation time fell to one quarter of the conventional process. The rotor–stator unit consumed 4.6 kWh/kg, lower than laboratory cavitation (7.4 kWh/kg) and conventional heating (15 kWh/kg). Relative to acoustic cavitation, hydrodynamic cavitation offered higher energy efficiency and superior performance in producing oil/water emulsions with smaller droplet sizes and improved stability. Consistently, Jasmina et al. [111] noted that ultrasound can produce nanoemulsions but faces limits: coalescence control, strong size sensitivity to settings, and poor scalability for industrial production.

**Fig. 7:**
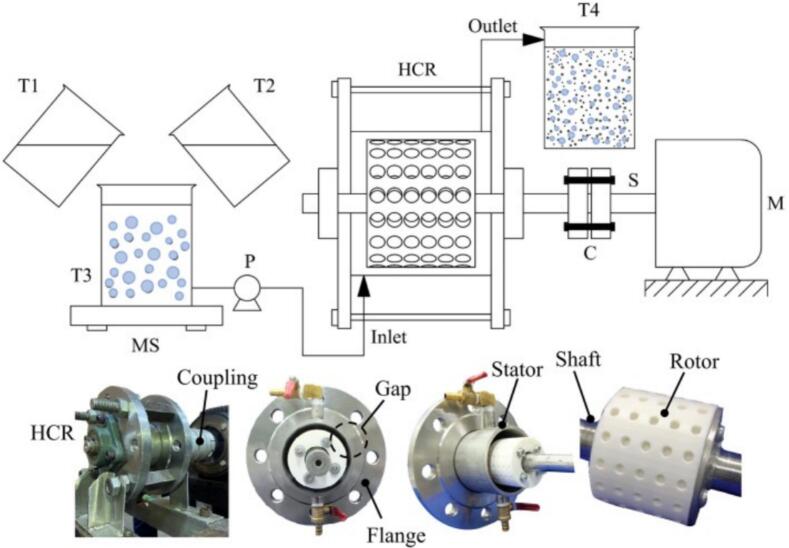
Emulsion preparation process and rotor characteristics [25].

**Fig. 8:**
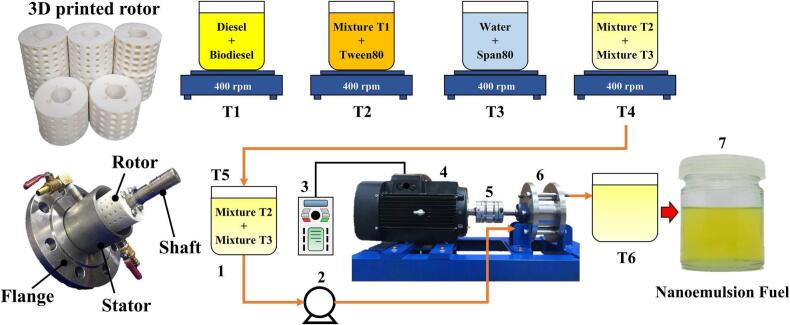
Preparation process of nanoemulsified fuel [61].

Driven by rapid growth in skincare markets, demand is rising for efficient and economical nanoemulsion production. Traditional methods are energy-intensive and hard to scale. Using a 3D-printed rotor (Fig. 7), Matman et al. [25] achieved continuous preparation of skincare nanoemulsions. Parameter and composition optimization yielded droplets of 366.4 nm, meeting cosmetic standards. Release tests showed the best performance for hydrodynamic-cavitation emulsions (366.4 nm/4335.8 μg cm^−2^ at 12 h) versus ultrasound (373.5 nm/4156.3 μg cm^−2^) and magnetic stirring (1879 nm/3545.4 μg cm^−2^). The emulsions were stable: after 90 days at ambient storage, size increased only to 491.7 nm, and freeze–thaw cycling caused no phase separation. These results demonstrate the feasibility of rotational cavitators for nanoemulsion skincare production. Using a similar 3D-printed rotor, Oo et al. [61] developed continuous preparation of diesel–biodiesel–water nanoemulsions (Fig. 8). A rotor with uniformly distributed spherical indentations produced an average size of 257.6 nm from a coarse emulsion of 844 nm under optimal settings (5.8 mm indentation diameter, 6.4 mm depth, 4011 rpm, 11.8 L/h). The nanoemulsions remained stable for >90 days at room temperature. Engine exhaust tests indicated lower NO_x_ emissions with acceptable fuel consumption and reduced exhaust temperature, suggesting environmental and engineering benefits for multiphase fuel applications.

**Fig. 9:**
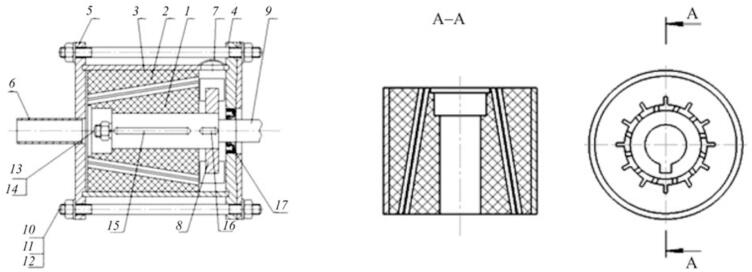
Schematic diagram of the axial rotational cavitator with a conical rotor [104].

**Fig. 10:**
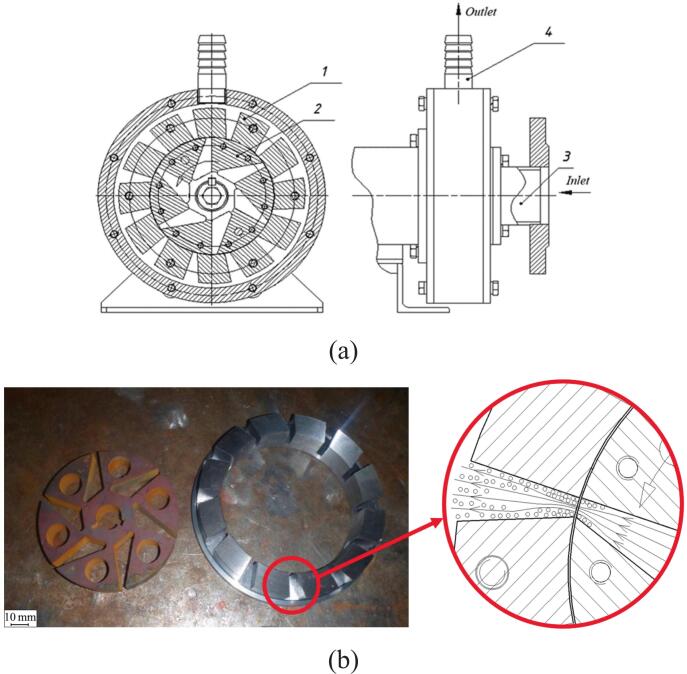
(a) Schematic of the radial serrated rotational cavitator; (b) Illustration of the rotor–stator structure and cavitation zone [106].

Tsymbalov et al. [104] used flow visualization to demonstrate the feasibility of rotational cavitators for producing highly dispersed oil-in-water (O/W) nanoemulsions. They evaluated rotor speed and surfactant type. As shown in Fig. 9, an axial cavitator with specially grooved conical rotor and stator replaces conventional recesses; the mixture passes through a narrow annular gap where shear and cavitation act together, yielding well-dispersed emulsions. Increasing speed from 3000 rpm to 6000 rpm reduced the mean droplet size from 2.42 μm to 1.93 μm. Focusing on geometry optimization, Omelyanyuk et al. [106] applied Computational Fluid Dynamics (CFD) to improve rotor–stator design (Fig. 10). A radial-serrated cavitator with asymmetric trapezoidal diverging rotor channels and converging stator channels creates overlapping shear and tangential stress in the transition zone, strengthening cavitation. Raising the speed from 15 Hz to 50 Hz increased the maximum static pressure and extended the cavitation zone length from 0.0009 m to 0.024 m. Proper frequency control thus lengthens the cavitation region, enhances mixing, and maintains pressure stability, providing guidance for design optimization and industrial deployment.

**Fig. 11:**
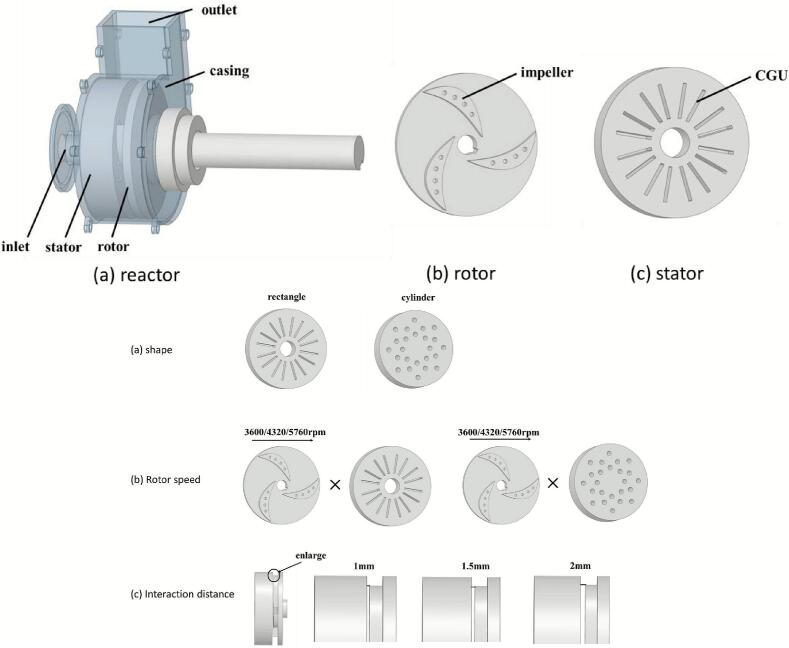
Structural schematic of the rotational cavitator featuring radial grooves [107].

Song et al. [107] proposed a radial-grooved rotational cavitator for emulsification and dispersion, and used CFD to study the effects of speed, rotor–stator gap, and internal geometry on cavitation (Fig. 11). Grooves on both rotor and stator trigger strong, periodic cavitation in the flow-separation zones. Higher rotor speed and a shorter interaction distance strengthen cavitation. Rectangular grooves outperform cylindrical ones in cavitation rate and energy efficiency; the latter restricts cavitation due to a smaller cross-section. When the gap increases from 1 mm to 2 mm, the cavitation rate falls from 1.695 % to 0.47 %. A shorter gap enhances shear-induced low pressure. Increasing speed from 4320 rpm to 5760 rpm raises the cavitation rate of rectangular grooves to 6.64 % and improves energy efficiency by 128 %. Building on these findings, Zhang et al. [108] evaluated cavitation performance and pressure pulsations using CFD with the *k–ε* turbulence model and Zwart cavitation model. Shear-driven bubbles generated by rotor rotation dominate within the rotor. At 4200 rpm, cavitation intensity increases markedly, and blade-frequency pulsation amplitudes rise along the flow path. These results guide design optimization and broaden applications in dispersion, emulsification, and process intensification. To move beyond a single cavitation mechanism, Zhao et al. [105] proposed a shear–ultrasound coupled continuous method. A coarse emulsion formed by high-speed shear enters an ultrasonic microreactor. With optimized settings, the droplet size reaches 307 nm. Dual-frequency operation (20 kHz and 40 kHz) further improves efficiency and energy use. After 30 days at room temperature, the size increases only from 307 nm to 331 nm (+7.8 %), indicating good mid-term stability. The approach offers low energy demand and high stability, with strong potential for continuous and scaled emulsification (see Table 5 for further details).

**Table 5:** Applications of rotational cavitators in droplet emulsification.

System	Parameters	Performance	Mechanism	Ref.
Paraffin O/W nanoemulsion	2000–2500 rpm; 17 L/min; SLS: 270.95 g	Droplet: ∼590 nm; stability >4 weeks; energy consumption reduced by ∼25 % vs ultrasonic	Alternating pressure induces jets and shear, breaking droplets and enhancing dispersion	[109]
WCO	90 °C	Reaction time shortened by 4×; stable emulsion	Bubble collapse generates turbulence, localized heating, and radicals, accelerating oxidation/polymerization	[110]
Oil–water	3500 rpm; 3.3 L/h; Span 60: 2.36 %; Tween 60: 3.00 %; mineral oil: 1.76 %	Droplet: 366.4 nm; size increased to 491.7 nm (90 days); improved nicotinamide release (4335.8 μg/cm2, 12 h)	Rotor–stator shear and vortex cavitation reduce droplet size, enhance mixing	[25]
Diesel–biodiesel–water	indentation diameter: 5.8 mm; depth: 6.4 mm; 4011 rpm; 11.8 L/h	Droplet: 257.6 nm; stable 90 days; NO_x_ emissions reduced by 60.4 % and 57.53 % (vs. diesel and biodiesel)	Shear and turbulence cause cavitation; bubble collapse releases heat/pressure, promoting mixing	[61]
Sunflower /Transformer oil–water	3000–6000 rpm; surfactants: EPL-1/SK-2	Droplet: 2.42 to 1.93 μm; stable emulsion	Rotor–stator shear + cavitation collapse enhance oil dispersion	[104]
Nanoemulsion (CFD)	Rotor: 15–50 Hz; inlet pressure: 1–10 atm; 25 °C; 10–40 L/min; *α* = 19.0 ± 0.5°, *β* = 20.0 ± 0.8°	Improved emulsification; reduced operating pressure; cavitation zone: 0.0009 m to 0.024 m	Channel expansion–contraction forms pressure gradients, intensifying cavitation	[106]
Nanoemulsion (CFD)	CGU rectangular; gap: 1 mm; 5760 rpm	cavitation probability increased to 6.64 %; energy efficiency improved by 128 %	Vortex cavitation in rotor–stator zone; bubble collapse enhances emulsification	[107]
Nanoemulsion (CFD)	4200 rpm; outlet velocity: 0.97 m/s; Zwart + Realizable *k-ε*	Periodic pressure fluctuation; outlet cavitation zones formed	Shear-induced vortex cavitation; bubble collapse improves mixing	[108]
Tween 80 + octane (1.0 wt% Span 80)	Dual-frequency ultrasound (20 + 40 kHz); 0.3 mL/min; circulation: 6;22,000 rpm; residence: 9.74 s	Droplet: 307 nm; low energy input; low surfactant content; high stability	Pre-shear forms coarse emulsion; 20 kHz cavitation breaks droplets, 40 kHz refines them	[105]

## 5 Food processing

**Fig. 12:**
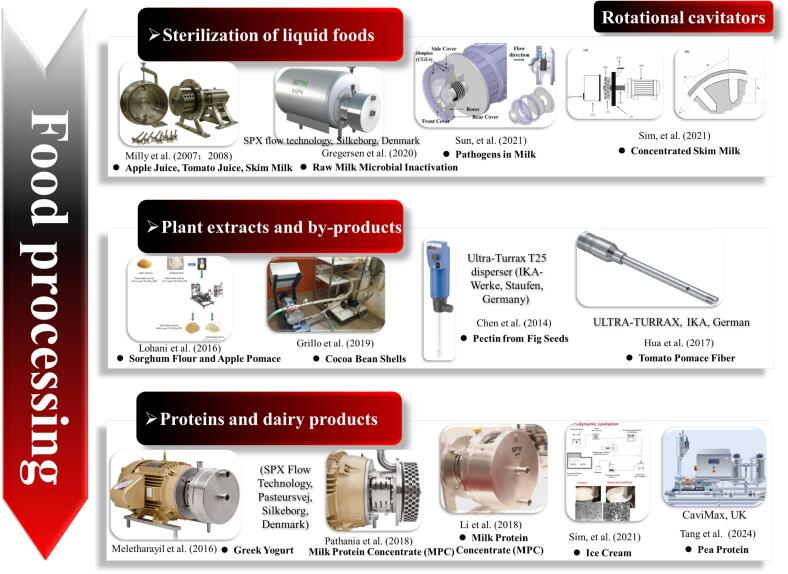
Evolution of rotational cavitator applications in food processing [60,65,115,118,[120], [121], [122], [123], [124], [125], [126], [127], [128]].

In the field of food processing, the rotational cavitator has emerged as a promising green technology, showing potential in key applications such as homogenization, extraction, sterilization, and rheological control (Fig. 12) [112,113]. The strong cavitation generated during operation produces localized high temperatures and pressures, intense shear forces, and micro-jets. These effects can effectively disrupt cell structures, promote the release of bioactive compounds, and significantly alter rheological properties, contributing to enhanced food waste valorization [114,115]. Cavitation also exerts microbial inactivation effects [116], enabling low-temperature processing while preserving nutritional and sensory quality [117]. The process is continuous and energy-efficient, meeting the demands of industrial-scale production and aligning with consumer preferences for additive-free and minimally processed foods [118,119].

### 5.1 Sterilization of liquid foods

**Fig. 13:**
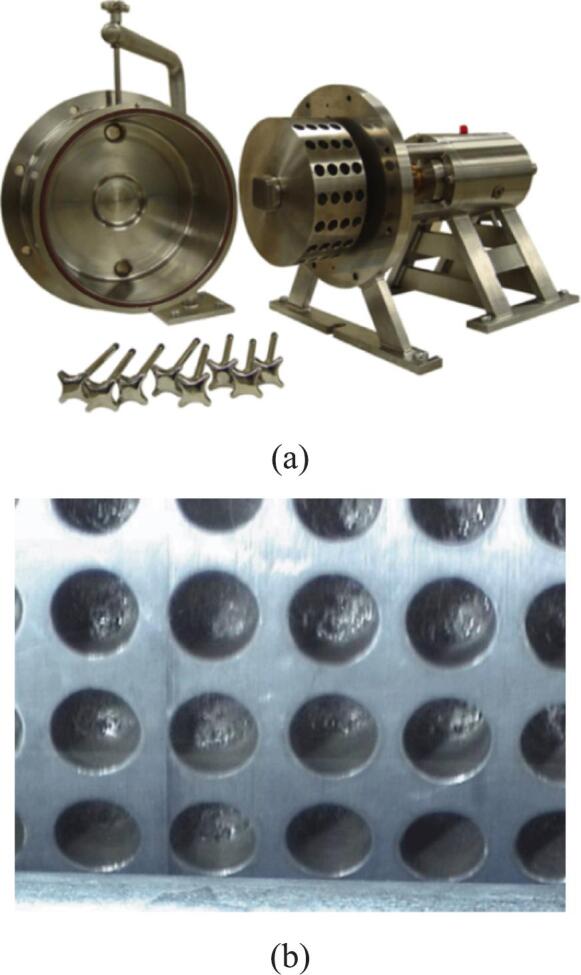
(a) Schematic diagram of the indentation-type rotational cavitator; (b) cavitation characteristics in the rotor indentations. [120].

Early studies by Milly et al. [120] evaluated rotational cavitators for fluid-food pasteurization using the setup in Fig. 13. Apple juice, tomato juice, and milk were treated at varied speeds and temperatures. The method showed strong inactivation of bacteria, yeasts, and spores. High-acid juices achieved 5-log reductions at moderate temperatures. For low-acid milk, spore inactivation was limited, indicating a need for design and residence-time optimization. The key mechanism was thermal–cavitation synergy, which enabled high lethality at lower temperatures. Building on this, Milly et al. [121] focused on apple juice. The device inactivated *Saccharomyces cerevisiae* at 65–76.7 °C, well below conventional pasteurization (88 °C), while cutting energy use and surpassing the thermal kill achievable by heat alone, with 55–84 % energy savings. These results show that rotational cavitation provides strong microbial inactivation under mild conditions, making it suitable for thermally sensitive juices.

**Fig. 14:**
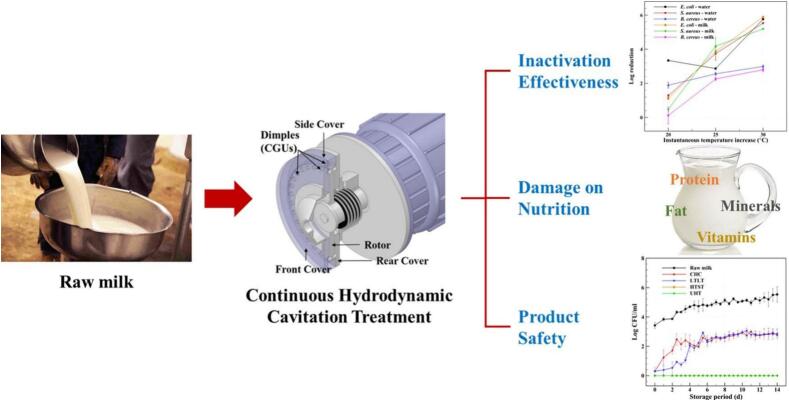
Process flow diagram for pathogen inactivation in milk [116].

Gregersen et al. [122] evaluated the APV Cavitator (SPX Flow Technology) for raw milk at different temperatures. The rotor had four rows of 40 cavitation indentations per row (single-indentation volume 8.04 cm^3^), providing stable bubble generation. Cavitation reduced fat globule size to 3.29–1.40 μm and improved homogenization without significant whey-protein denaturation or impairment of acid gel formation. Total viable counts fell by >1 log, mainly due to process heating. Compared with conventional thermal treatment, hydrodynamic cavitation achieved simultaneous disinfection and homogenization while better preserving milk properties. Similarly, Sun et al. [116] applied rotational cavitator (Fig. 14) to pathogen inactivation in milk and assessed thermal behavior, lethality, nutrient retention, safety, and cost. At 70 °C for 1–2 s, the system achieved >5-log inactivation of *E. coli* and *S. aureus* with minimal nutrient loss, comparable to high-temperature short-time (HTST) pasteurization; refrigerated at 5 °C for 14 days, microbiological stability matched low-temperature long-time pasteurized milk. The mechanism involves mechanical disruption (membrane collapse), thermal effects (protein denaturation), and radical oxidation (^•^OH). The device offered low cost ($0.00268 L^−1^) and high throughput (4.2 L/min), indicating potential for continuous processing of liquid foods.

Under 35 % total solids, heat treatment alone achieved only a 2.77 log CFU/mL reduction. Using a rotational cavitator, Sim et al. [123] increased inactivation to 3.5 log CFU/mL and shortened the treatment time. The device (SPX Flow Technology, Crawley, UK) comprises a fixed housing and a rotor with 88 equally spaced cylindrical indentations and operates up to 3600 rpm (see Table 6 for further details).

**Table 6:** Applications of rotational cavitators in sterilization of liquid foods.

Substrate	Parameters	Performance	Mechanism	Ref.
Fluid foods (apple, tomato juice, milk)	3000–3600 rpm; 65.6–115.6 °C	total process lethality: >5 log reduction in apple juice (65.6–76.7 °C); 3.10 log in *B. coagulans* (tomato juice, 104.4 °C); 2.84 log in *C. sporogenes* (milk, 115.6 °C)	Cell wall destruction by mechanical shear, pressure fluctuation, and localized heating enhances microbial inactivation	[120]
Apple juice (*S. cerevisiae*)	3000–3600 rpm; 65.6–76.7 °C	6.27 log CFU/mL reduction; lower energy consumption (conventional thermal treatment 258 kJ/kg)	Bubble collapse generates shear/thermal effects disrupting yeast cells	[121]
Raw milk	3300 rpm; 400 L/h; 40–72 °C	Fat globules reduced to 1.40–3.29 μm; >1 log microbial reduction; minimal effect on quality	Cavitation-induced heating + mechanical breakup of fat globules	[122]
Milk pathogens (*E. coli*, *S. aureus*, *B. cereus*)	3600 rpm; 70 °C; duration: 1–2 s	Log reductions: 5.89 (*E. coli*), 5.53 (*S. aureus*), and 2.99 (*B. cereus*); nutrient retention similar to HTST; stability comparable to long-time low-temperature (LTLT)	Combined mechanical, thermal, and ^•^OH radical effects	[116]
Concentrated skim milk	3600 rpm; 100 L/h; 75–85 °C; 14–106 s	3.5 log CFU/mL reduction vs. 2.77 log from heat alone	Cavitation weakens spore structures, enhancing thermal lethality	[123]

### 5.2 Plant extracts and by-products

**Fig. 15:**
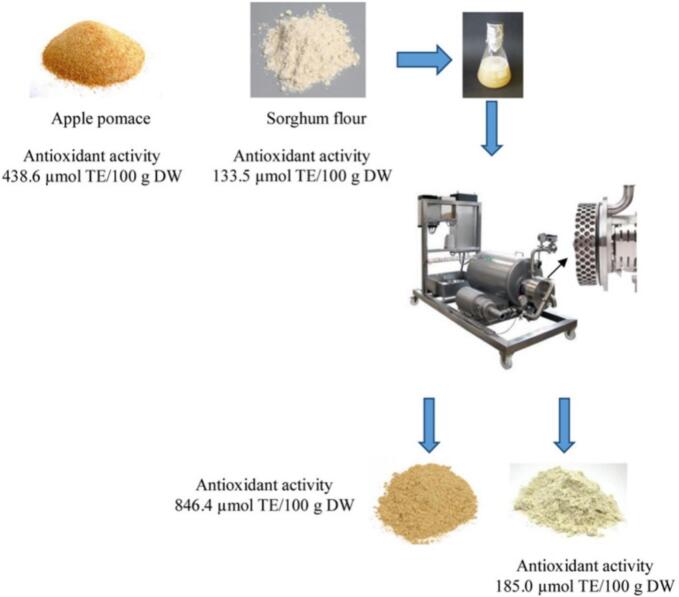
Flowchart of the combined fermentation and cavitation treatment process [115].

Ashokkumar et al. [129] reviewed advances in acoustic cavitation for food processing, with emphasis on physicochemical mechanisms. Industrial deployment remains limited by reactor design. To address this, the authors proposed hydrodynamic cavitation as a scalable alternative and outlined a design model for a rotational cavitator. Targeting the energy and scale limits of ultrasound, Lohani et al. [115] combined natural fermentation with a rotational cavitator to release bound phenolics from sorghum flour (SF) and apple pomace (AP). Operating variables—solid–liquid ratio, number of rotor-indentation rows, and temperature—significantly affected total phenolic content (TPC) and antioxidant activity (AA). Under optimized conditions (Fig. 15), SF showed +39.5 % TPC and +38.6 % AA, while AP reached +42 % TPC and +97 % AA. Rheology differed: SF was shear-thinning, whereas AP was shear-thickening. scanning electron microscope (SEM) images confirmed cell disruption and porous structures after fermentation plus cavitation, facilitating phenolic release and highlighting potential as an alternative to ultrasound. Grillo et al. [118] treated cocoa bean shells with rotational cavitator. Compared with ultrasound, it achieved efficient cell rupture and compound release at lower energy. Yields were 20.5 % (w/w, hydrophilic) and 15.8 % (w/w, lipophilic). The hydrophilic extract was rich in antioxidants (TPC 197.4 mg/g). Pre-milling and sieving reduced total fiber and enriched target compounds; removed fibers could serve as feed or mulch. The lipophilic extract contained 96.4 % fatty acids (w/w), closely matching commercial cocoa butter and indicating potential as a food lipid substitute.

Pectin readily aggregates in aqueous media, complicating structural analysis. Chen et al. [124] examined the effects of high-speed rotor–stator shear (24,000 rpm) on depolymerization and degradation of fig-seed pectin. During operation, the medium is drawn in axially and expelled radially at high velocity through the rotor–stator gap. Depolymerization dominated at short times, whereas degradation prevailed with longer treatment. Spectroscopic analysis indicated that the pectin backbone remained intact, confirming a combined physical–chemical action of mechanical shear. Similarly, Hua et al. [60] compared high-speed homogenization (HSH) and high-pressure homogenization (HPH) for tomato-pomace fibers. A rotational cavitator markedly altered microstructure: large particles (>120 μm) were reduced to ∼60 μm, and soluble fiber increased by ∼8 %. The enhancement is attributed to a larger specific surface area, shear-driven diffusion and collisions, and shear-induced thermal effects (see Table 7 for further details).

**Table 7:** Applications of rotational cavitators in plant extracts and by-products.

Substrate	Parameters	Performance	Mechanism	Ref.
Sorghum flour, apple pomace	Sorghum: 100 g/L, 3-indentation-array, 35 °C; apple pomace: 87.5 g/L, 4-indentation-array, 45 °C	Total phenolic content increased by 39.5 % (sorghum) and 42 % (apple); antioxidant activity was enhanced by 38.6 % and 97 %	Bubble collapse and localized heating disrupt cells, releasing bound phenolics	[115]
Cocoa bean shells	(water/ethanol/hexane); 3000 rpm; 11 min	Extract yields: 20.5 % (hydrophilic), 15.8 % (lipophilic); hydrophilic TPC 197.4 mg/g; lipophilic fraction ∼96.4 % fatty acids	Shockwaves and microjets(bubble collapse) enhance solid–liquid contact and disrupt biomass	[118]
Fig seed pectin	24,000 rpm; 24 h	Pectin molecular weight: 2566.3 to 212.7 kDa; reducing sugar: 1.5 × higher (vs. 8 h)	Shear breaks aggregates and glycosidic bonds without backbone cleavage	[124]
Tomato pomace fiber	HSH: 15000 rpm/2 min; HPH: 100 MPa/10 cycles	Soluble fiber increased by 8 %; water- and oil-holding capacities improved	Shear/pressure disrupt fibers, release microfibers; acidic pH aids degradation	[60]

### 5.3 Proteins and dairy products

In dairy processing, rotational cavitators can disrupt fat globules, modify rheology and homogenization, and preserve nutritional and functional attributes. Meletharayil et al. [125] combined CO_2_-treated milk protein concentrate (TMPC) with rotational cavitation for Greek-style yogurt. Energy release altered protein–protein interactions, reduced particle counts, and improved texture. The product outperformed commercial samples in acidity control, viscosity, microstructure, and water-holding capacity, offering a route to replace centrifugal whey removal. To improve reconstitution of milk protein concentrate (MPC), Pathania et al. [126] compared conventional shear with a rotational cavitator. After high-speed shear, many undissolved particles remained (*D*_90_ = 21.17 μm; *D*_[__4__,__3__]_ = 5.62 μm). Cavitation reduced sizes to 0.45 μm and 0.19 μm, lowered apparent viscosity, and improved anti-sedimentation during storage. The effect stems from bubble-collapse shear that disrupts casein micelles and accelerates wetting, penetration, dissolution, and swelling. Li et al. [127] showed further benefits before spray drying. Using an SPX rotational cavitator, disruption of protein gels reduced elasticity and viscosity of high-solids emulsions; at 25 Hz and 50 Hz, viscosity dropped by ∼20 % and ∼56 %, respectively. Solubility was unaffected (average 97.5 % at 50 °C), while smaller droplet sizes increased bulk and tapped densities of the powder. Beyond microbial inactivation, Sim et al. [65] explored low-stabilizer ice cream at pilot scale (2400–3600 rpm; 100–200 L/h). Cavitation shifted the mix from a viscoelastic solid to a viscoelastic liquid and raised high-shear viscosity to ∼2.2× the control. These changes in rheology further influenced the melting behavior of the final ice cream, manifesting as structural collapse and fat agglomeration. This tunable rheology supports reduced-additive formulations. Recently, Tang et al. [128] reported pea-protein extraction with cavitation; rotational cavitation scaled well and increased protein purity (80.35 g/hg) and recovery (56.85 g/hg) over conventional methods. Structural analyses indicated intact peptide bonds with changes in secondary and tertiary structures, and SEM showed less protein retained in residues, confirming higher extraction efficiency (see Table 8 for further details).

**Table 8:** Applications of rotational cavitators in proteins and dairy products.

Substrate	Parameters	Performance	Mechanism	Ref.
Greek-style yogurt	3490 rpm	Viscosity decreased (TMPC yogurt *η* = 0.25 Pa·s, MPC yogurt *η* = 0.66 Pa·s), and particle number: 13–35/g, approaching the commercial quality	Shear and bubble collapse disrupted protein networks, reducing particles and improving texture	[125]
MPC	2914 rpm; 850 L/h; back pressure: 2.4 bar; 50 °C	Full rehydration; *D*_90_: 21.2 to 0.45 μm; viscosity decreased and stability improved	Cavitation shear/turbulence disrupted micelles, promoted wetting, solubilization, and dispersion	[126]
MPC	Rotor: 25 or 50 Hz; 100 L/h	Viscosity decreased by 20–56 %; particle size reduced, density increased; solubility (50 °C) reached 97.5 %	Shockwaves (cavitation) disrupted gel networks, improving flow without denaturation	[127]
Ice cream mix	2400–3600 rpm; 100–200 L/h	Particle size: 3.52 ± 0.28 μm and 0.34 ± 0.02 μm; rheological behavior: viscoelastic solid to liquid, and viscosity increased 2.2 × under 30–50 Hz	Cavitation shear/turbulence modified particle size and rheology, enhancing stability	[65]
Pea protein	Rotor: 50 Hz; 800 L/h	Purity: 80.35 g/hg; recovery: 56.85 g/hg	Cavitation microjets/shear disrupted cells and altered higher-order structures, enhancing extraction while preserving primary structure	[128]

## 6 Wastewater sludge

**Fig. 16:**
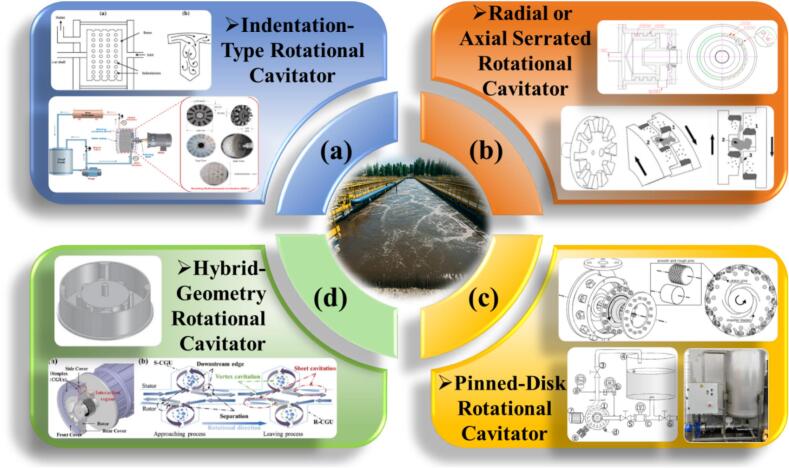
Types of cavitators used in wastewater sludge treatment; (a) indentation-type rotational cavitator [62,135]; (b) radial or axial serrated rotational cavitator [136,137]; (c) pinned-disk rotational cavitator [138,139]; (d) hybrid-geometry rotational cavitator [140,141].

Cavitation has gained traction in wastewater and sludge treatment for its ability to enhance mass transfer, accelerate organic degradation, and raise overall efficiency [12,130]. Among enhancement routes, rotational cavitators stand out: high-speed rotation generates localized cavitation with flexible operation, low energy demand, and good scalability [131,132]. The resulting high-energy microenvironment disrupts recalcitrant structures, promotes redox reactions, and improves biodegradability. Notably, as a purely physical process, it achieves effective and environmentally friendly disinfection without the need for additional chemical reagents [57]. Compared with conventional hydrodynamic or ultrasonic systems, rotational cavitation shows superior adaptability for integrated cell disruption, particle disintegration, and organics removal [19,133]. Current studies commonly combine experimental investigation with numerical simulation to explore cavitation mechanisms, while optimizing structural designs to enhance cavitation intensity [134]. As shown in Fig. 16, various types of rotational cavitation devices have been developed for wastewater sludge disinfection, including indentation-type, radial or axial serrated, and pinned-disk designs. Several novel configurations have also emerged based on these foundations.

**Fig. 17:**
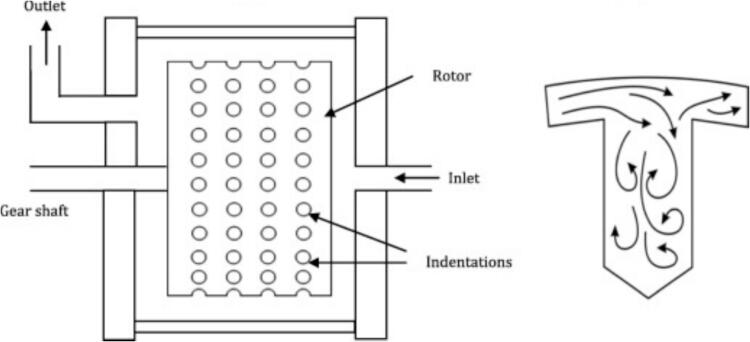
Configuration of the cavitation device and internal flow behavior within the indentations [62].

In early studies, Indian researchers Jyoti and Pandit [142] first verified the feasibility of rotational cavitation for drinking-water disinfection as a non-chemical, energy-efficient method that avoids toxic by-products. At 8000 rpm for 15 min, bacterial removal reached 92 % with a high energy yield. Applying the approach to wood-finishing wastewater, Badve et al. [62] used an indentation-rotor cavitator (Fig. 17) and showed that bubble-collapse shear and shock waves cleave macromolecules into more oxidizable intermediates, while ^•^OH from water splitting enhances degradation; optimizing speed, H_2_O_2_ dose, and residence time markedly increased chemical oxygen demand (COD) removal, with cavitation yield improving by 46 % under best conditions. For algal control, Maršálek et al. [143] found that the indentation-rotor cavitator alone achieved <40 % cyanobacteria inhibition, whereas coupling with ultra-low H_2_O_2_ enabled 99 % removal in a single 6-second pass. This efficiency, far exceeding conventional hydrodynamic cavitation, was attributed to combined vortical cavitation in rotor bores and shear cavitation in the rotor–stator gap. In pharmaceutical effluents, Mukherjee et al. [135] degraded ciprofloxacin to 44.8 % in 60 min by cavitation alone and to 85.6 % in 30 min with 0.3 g/L H_2_O_2_, identifying this as the best energy–cost trade-off and confirming feasibility in pilot trials. Extending to triclosan, Mishra et al. [144] found that cavitation alone—optimized at 2700 rpm—achieved only 35.2 % removal, indicating its limited oxidative capacity for persistent pharmaceuticals. When combined with ozone, removal increased sharply to 97.6 %, showing that cavitation functions more as an intensification step rather than an effective standalone treatment.

**Fig. 18:**
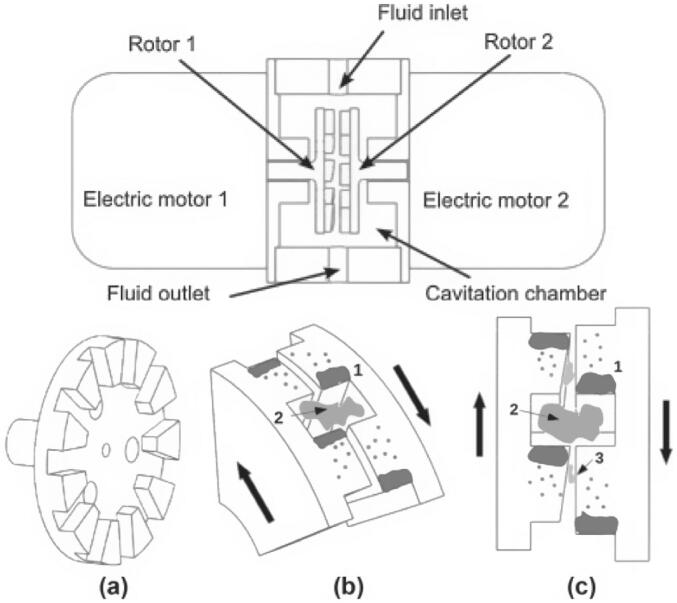
Structural schematic of the cavitation device (left) and serration configurations (right): (a) perpendicular-edge rotor, (b) perpendicular serration pair, and (c) 8° inclined serration pair [137].

Petkovšek et al. [137] proposed and validated a radial-serrated rotational cavitator to remove pharmaceuticals from water (Fig. 18). The unit consists of two counter-rotating rotors with radial grooves; the inter-rotor gap forms a Venturi-like passage. Periodic pressure pulsations initiate cavitation in the gap, and the rotors drive bubbles into the grooves where shear cavitation develops. An 8°-inclined rotor produced a larger cavitation zone, stronger pressure fluctuations, and more aggressive erosion than a right-angle rotor. Cavitation combined with H_2_O_2_ markedly improved removal, whereas cavitation alone was limited. Degradation proceeds via radical generation, which depends strongly on temperature and H_2_O_2_ dosage. Building on these results, Zupanc et al. [145] treated additional pharmaceuticals. In deionized water, cavitation combined with H_2_O_2_ achieved 47–86 % removal; however, careful control of the H_2_O_2_ dosage was required to avoid inhibition of degradation. In real wastewater, matrix effects reduced performance, but higher oxidant doses and longer residence times compensated for the loss. As a pretreatment, the cavitator enhanced diclofenac and carbamazepine removal to 54 % and 67 %, outperforming 39 % and 56 % as a post-treatment. Addressing contaminants of emerging concern (CEC), Kovačič et al. [146] assessed the same device for 46 compounds. At 9500 rpm and 10 min, removals for several bisphenols reached 63 %, with strong dependence on physicochemical properties and temperature. Pilot-scale tests showed that lower rotor speeds not only reduced energy use but also increased removal, up to 90 %.

**Fig. 19:**
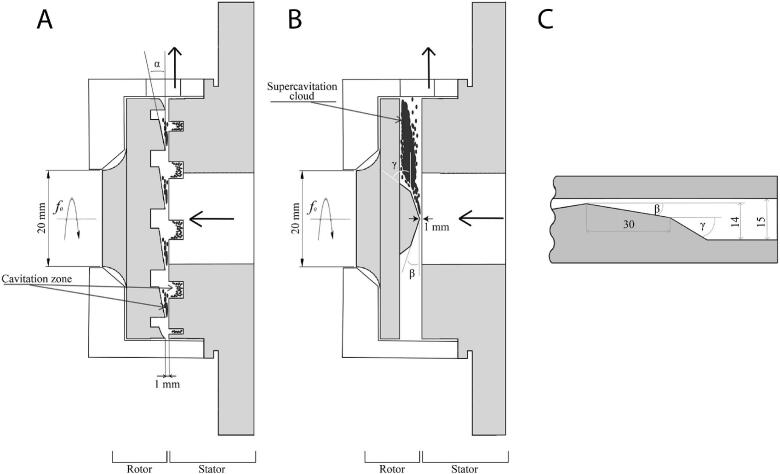
Cavitation device configurations [148]: (A) rotor–stator pair generating developed cavitation [137]; (B) rotor–stator pair generating supercavitation [147]; (C) conventional Venturi-type constriction

Šarc et al. [147] designed a novel rotational cavitator for water disinfection and showed strong pathogen inactivation. A Venturi-like constriction between the rotating serrations and the stator induces tangential and radial flows. Fluid accelerates past serration tips, local pressure drops, and cavitation forms. Large inter-serration spaces sustain a stable supercavity. Comparing regimes—incipient, developed, and supercavitation, the study found that only supercavitation achieved >3-log reductions of *Legionella* and *E. coli*, with additional action against Gram-positive bacteria. Performance and cost were superior to Venturi cavitators; the mechanism likely involves rapid pressure collapse as cells enter the supercavity. Extending this work, Kosel et al. [148] tested two devices (Fig. 19): Petkovšek’s serrated-rotor unit generated developed cavitation (unsteady bubble clouds and pressure pulsations), while Šarc’s dual-serration rotor formed a stable supercavity. In real wastewater, 1 h of supercavitation reduced yeasts, anaerobic sulfate reducing bacteria, and aerobic bacteria by 3–4 logs, lowered COD by 22 %, and cut insolubles and deposits by >50 % with 1/4 the energy of a Venturi unit.

Building on work in pharmaceutical removal and disinfection, Sežun et al. [149] applied a radial-serrated rotational cavitator to valorize secondary sludge from the paper industry. Compared with conventional units, the device increased the release of soluble COD, total nitrogen, and total phosphorus. With NaOH alkali, 30 min of treatment raised COD_s_ and total nitrogen to 2400 mg/L and 120 mg/L, respectively, and microscopy showed clear floc disintegration. The combined cavitation–alkali route enhanced sludge processing at low cost—about €1 per 1.9 kg COD_s_ released. Arteaga et al. [150] further coupled ethylenediamine tetraacetate (EDTA) washing with hydrodynamic cavitation to remove multiple heavy metals and enrich plant-available phosphorus, indicating agricultural potential. Ren et al. [151] investigated a radial-serrated device using experiments and CFD. The system employs counter-rotating rotors with inclined and flat serrations. Cavitation clouds varied cyclically between the interacting rotors. Two pressure-pulsation periods matched changes in cavitation volume fraction, and blade-passing frequency was identified via Fourier analysis.

**Fig. 20:**
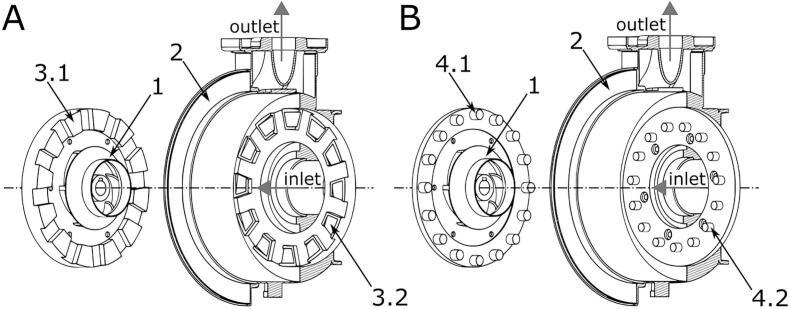
Comparison of cavitator designs: (A) conventional radial serrated rotor; (B) novel pinned-disk rotational cavitator [138].

Building on radial-serrated designs, Gostiša et al. [138] developed and validated a pinned-disk rotational cavitator (Fig. 20). It delivered higher cavitation intensity and better energy efficiency than serrated units. In wastewater treatment, enhanced mechanical shear and oxidative action improved particle properties. The advantage stems from more favorable rotor–stator flow conditions; by contrast, serrated rotors suffer stronger vortical and shear losses, weaker cavitation, and limited ^•^OH generation and COD removal. Under optimal settings, COD removal increased by 310 % and energy use fell by 65 % relative to serrations, indicating strong engineering potential. In a follow-up study, the same group [139] analyzed pin geometry and layout. Rotor speed and pin spacing were the key factors for cavitation strength; pin diameter and surface roughness had minor effects, and overly dense pin arrays suppressed cavitation. With an optimized layout, 15 recirculations yielded 31 % COD removal at 8.2 kWh/kg, highlighting the role of pass number in energy performance. Repinc et al. [152] further showed that pinned-disk cavitator reduced sludge particle size by 88 %, tripled specific surface area, enhanced organics solubilization, and lowered Pb content. Microscopy confirmed cell disruption without DNA release.

**Fig. 21:**
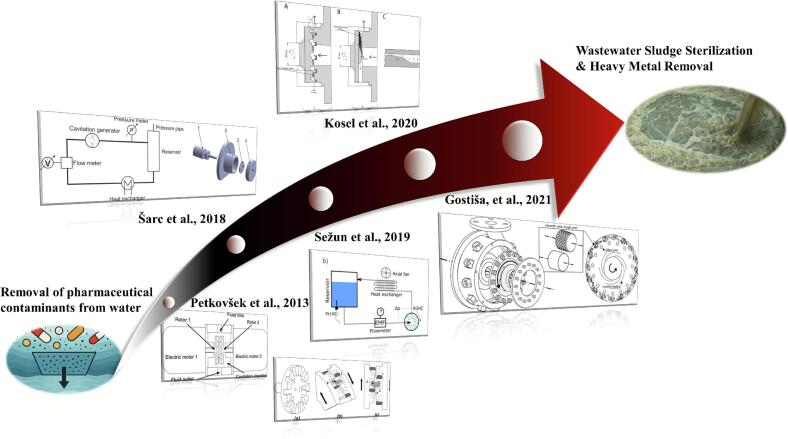
Schematic illustration of cavitator structural evolution (from pharmaceutical removal in water to wastewater sludge treatment) [137,138,[147], [148], [149]].

To systematically illustrate the structural evolution and application advancements of the aforementioned rotational cavitators, a schematic diagram (Fig. 21) has been developed. This figure outlines the progression from early configurations, such as radial serrated designs, to advanced structures like the pinned-disk cavitator. It highlights key structural improvements and offers a clear visual reference for understanding the technological evolution and engineering adaptability of various cavitator types.

**Fig. 22:**
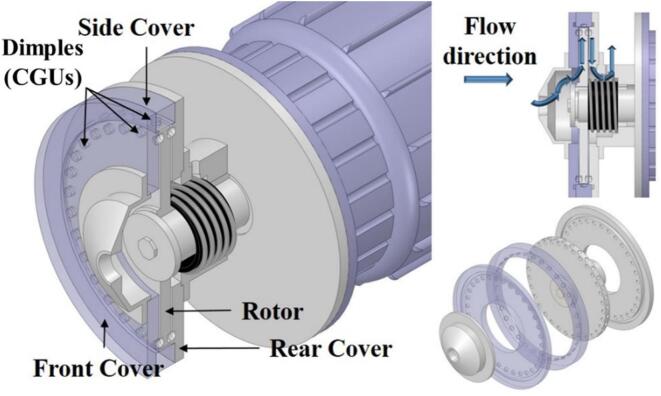
Structural schematic of the hybrid-geometry rotational cavitator [66].

Focusing on the often-overlooked thermal role in disinfection, Sun et al. [153] built a rotational cavitator with indentations on both rotor and stator, which strengthened cavitation versus rotor-only designs. Flow visualization, calorimetry, and bioassays showed up to 48.15 MJ/h heat release and 82.18 % thermal efficiency. At 3600 rpm and 0.5 bar, *E. coli* was efficiently inactivated, with thermal effects dominating. At 11 L/min for 14 min, disinfection reached 100 %, about 69 × faster than conventional heating; the mechanism combines thermal action, mechanical impacts, and radical oxidation. Under equal energy input, Kim et al. [19] found hydrodynamic cavitation outperformed ultrasound in particle breakage and organic oxidation, while solubilization was similar (42.3 % vs 41.4 %). Kim et al. [154] further showed that indentations and thermal effects boosted sludge disintegration by 50–80 %; fragmentation increased with speed but weakened at high inlet pressure. Further, with a side-mounted multi–cavitation-unit rotor [66], vortex and sheet cavitation coexisted and a critical flow rate emerged; thermal efficiency rose to 79.99 %, and *E. coli* was fully removed at $2.72/m^3^, delivering ∼140× higher throughput at ∼1/50 the cost of conventional systems (Fig. 22). In follow-up work, Sun et al. [57] confirmed superior processing rate and energy efficiency; SEM revealed severe cell damage, supporting a coupled hydrodynamic–sonochemical inactivation pathway.

**Fig. 23:**
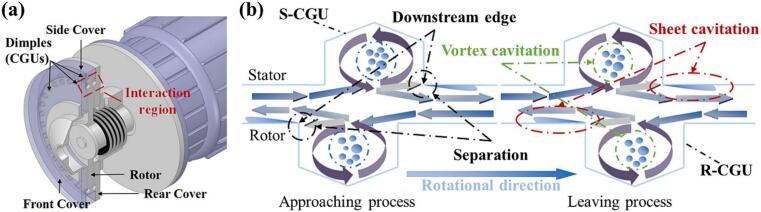
Flow structures and cavitation mechanisms in the rotor–stator interaction region [141].

To resolve flow structures, cavitation mechanisms, and their interactions, Sun et al. [155] combined experiments with CFD. They divided the cavitation evolution into three phases: overlap, separation, and approach. The behavior was periodic with a 0.5 ms cycle. Sheet cavitation formed downstream of the rotating and stationary cavitation-generating units (CGUs), expanded during separation and approach, and collapsed during overlap. Vortex cavitation arose inside the CGUs and was shaped by compression due to CGU interaction. Using a simplified-flow CFD framework, Sun et al. [156] then assessed CGU layout effects. Key parameters were the radial offset (*c*), crossing angle (*ω*), number of rows (*N*), circumferential offset (*γ*), and radial spacing (*r*). Small *ω* and moderate settings improved cavitation efficiency, although side-wall effects limited the simplified model. Building on this, Xia et al. [141] used the *Q*-criterion and vorticity-transport theory to reveal vortex–cavitation coupling in the device (Fig. 23). CGU-induced spiral flows promoted sheet and vortex cavitation. Vortices clustered within and along CGU edges and evolved dynamically. Two types were identified: columnar vortices, which periodically detached from wall vortices, and conical vortices formed by flow convergence along CGU walls. Vorticity transport was dominated by stretching and dilatation, while the baroclinic term concentrated at the vapor–liquid interface due to strong density and pressure gradients.

**Fig. 24:**
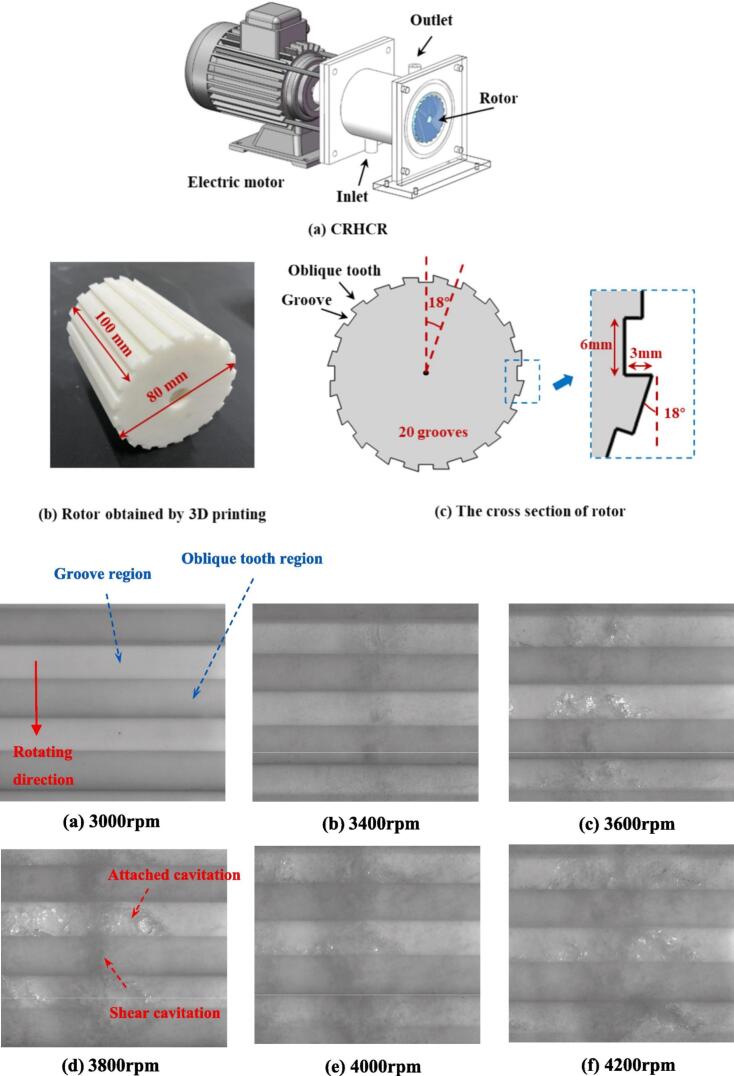
Rotational cavitator schematic and cavitation behavior under varying speeds [159].

In addition, Cerecedo et al. [140] used TEM to show bacterial damage and attributed inactivation to combined shear, micro-jets, and ^•^OH radicals. The rotational cavitator was 1–2 orders of magnitude more energy-efficient than conventional units, and geometry optimization (rotor diameter, channel contraction ratio, blade number) further improved cavitation and disinfection. With the rise of axial-serrated designs, Vilarroig et al. [157] evaluated pretreatment of activated-sludge/pig-slurry mixtures at lab and industrial scales; scale-up enhanced energy efficiency (specific energy of the sludge solubilization and specific energy dropped by ∼one order of magnitude) and achieved a maximum disintegration degree (DD) of 17 %. Fu et al. [158] arranged rotor–stator serrations helically with two lead angles for wastewater treatment; both designs strengthened cavitation and internal flow, while the higher-lead variant increased bubble counts by 54 % and delivered higher throughput. Processing capacity decreased with increasing Re. Lyu et al. [136] induced cavitation by staggered motion of moving and stationary serrations, enabling continuous cavitation and high throughput; CFD with the Zwart model showed strong dependence on structure, with the number of serration rows most critical—eight rows formed a stable low-pressure core and the best cavitation, whereas too many serrations in a single row restricted flow. Larger outer diameter enhanced centrifugal and shear effects; serration inclination optimized bubble nucleation sites. Recently, Xue et al. [159] proposed an axial cylindrical cavitator with rectangular grooves and oblique serration protrusions (Fig. 24). High-speed imaging revealed attached cavitation (trapezoidal clouds in grooves; inception–growth–collapse) and shear cavitation in the rotor–stator chamber with pulsation frequency linearly scaling with speed. Their synergy markedly improved cavitation performance (see Table 9 for further details).

**Table 9:** Applications of rotational cavitators in wastewater sludge.

Target	Parameters	Performance	Mechanism	Ref.
Groundwater (bacteria)	8000 rpm; treatment: 15 min	92 % removal	Bubble collapse ruptures cells; shear and heat enhance inactivation	[142]
Wood finishing wastewater (COD 38,000 mg/L)	2200 rpm; H_2_O_2_: 5 g/L; time: 20 min	49 % COD reduction (89 % with H_2_O_2_)	Shear/shock break macromolecules; ^•^OH from H_2_O_2_ accelerates oxidation	[62]
Cyanobacteria	5000 rpm + 45–100 μM H_2_O_2_; single: 6 s	99 % removal	Shear and microjets rupture cells; radicals selectively oxidize biomass	[143]
Ciprofloxacin (CIP)	2700 rpm; pH: 2; H_2_O_2_: 0.3 g/L; 120 cycles	44.8 % removal (85.6 % with H_2_O_2_)	Cavitation + H_2_O_2_ produce ^•^OH radicals, accelerating degradation	[135]
Triclosan (TCS)	2700 rpm; pH: 2; O_3_: 0.75 g/h; 120 cycles	35.2 % removal (97.6 % with O_3_)	Bubble collapse generates radicals; synergy with ozone drives oxidation and thermal decomposition	[144]
Pharmaceutical (ibuprofen, ketoprofen)	8° rotor; 100 kPa; 60 °C; 15 min; H_2_O_2_: 10 mL/L	Up to 82 % removal (4 drugs)	Shear cavitation + H_2_O_2_ generate ^•^OH radicals, degrading pharmaceuticals	[137]
Pharmaceutical residues (municipal wastewater)	Deionized water: 50 °C, 0.34 g/L H_2_O_2_, 15 min; Real water: 50 °C, 3.4 g/L H_2_O_2_, 30 min	47–86 % removal (real: 37–79 %)	Bubble collapse + H_2_O_2_ radicals degrade drugs (diclofenac, carbamazepine)	[145]
Bisphenols and emerging contaminants	Lab: 9500 rpm, 10 min; pilot: 2290 rpm	Bisphenols: up to 63 %; others: 15–90 %	Cavitation clouds collapse, producing heat/pressure and ^•^OH radicals	[146]
Pathogenic (*Legionella*, *E. coli*, *B. subtilis*)	0.2 L/min (H = 10 m)	3.3–3.8 log reduction (99.95–99.98 %)	Supercavitation cavities rupture membranes via pressure drop and shear	[147]
Paper industry process water	10,000 rpm; 60 min; 0.2 L/min	Anaerobic sulfate reducing bacteria and yeast: 4 log reduction, aerobic bacteria: 3 log reduction; COD: 22 % reduction, redox potential: 37 %. increase	Supercavitation cavities generate pulses/shear destroying microbes; ^•^OH minor role	[148]
Secondary pulp mill sludge	NaOH (30 min), 2800 rpm	COD_s_: 2386 mg/L, total nitrogen (Nt): 120 mg/L, Superior to monotreatment	Shear breaks flocs; NaOH + cavitation enhance hydrolysis/oxidation	[149]
Wastewater sludge (heavy metal)	10000 rpm, 30 min, EDTA: 50 mmol/L, citric acid: 50 mmol/L	Pb 35 %, Zn 68 %, Cd 47 %, Cu 45 %(removal); phosphorus increased 3.3 times	Shear disperses flocs; local heating + acid assists dissolve metal oxides	[150]
Ballast water microbes	gap: 1 mm; 4200 rpm	Cavitation rate: 500 % increase; energy density by 475 % increase	Counter-rotors form *Q*-vortices and low-pressure zones	[151]
Wastewater (particles + organics)	2700 rpm; 30 cycles; 8.6 L/s	Particle refinement; COD removal 310 % higher than that of serrated disc (SD); energy 65 % lower	Shear breaks particles, radicals oxidize organics; pinned-disk structure boosts efficient cavitation	[138]
Municipal wastewater	8 rotor pins (10 mm); 3000 rpm	COD removal 31 % (15 recirculations), energy:8.2 kWh/kg COD	Optimal pin spacing enhances turbulence and cavitation; excess pins suppress	[139]
WAS	Layout A: pin diameter: 16 mm/2700 rpm; B: pin diameter 10 mm/3000 rpm; 15–30 cycles	Size 88 % reduction; surface: 300 % higher; SCOD increased 155.8 %; Pb decreased 70 %	Unsteady cloud cavitation drives shock, heat, and oxidation	[152]
Simulated wastewater (*E. coli*)	3600 rpm; 11 L/min; 0.5 bar; 14 min	100 % inactivation (64.5 °C after 14 min)	Microjets + shear + heat + ^•^OH radicals synergize	[153]
WAS	Four energy configurations	Solubilization 42.3 %; better than ultrasonic	Cavitation (physical: particle/microbe breakdown; thermal: cell disruption) and radicals (H-bond cleavage) inactivate microbes	[19]
Sludge degradation	Pressure: 0.2–1.0 bar; 2100–3000 rpm	size reduced 50–80 %; SCOD significantly increased	Physical shear + thermal collapse dominate decomposition	[154]
Simulated wastewater (*E. coli*)	4200 rpm; 2.0 m^3^/h	100 % removal in 4 min; $2.72/m^3^; 140 × faster, 50 × cheaper	Vortex + sheet cavitation produces shock/heat/radicals	[66]
Wastewater (*E. coli* removal)	4200 rpm; 1.4 m^3^/h	100 % removal in 4 min; 3.75 L/min; 0.0499 kWh/L	Hydrodynamic + sonochemical effects disrupt microbes	[57]
Water treatment	3600 rpm; 1.4 m^3^/h	Sheet cavitation formed periodically and collapsed during the coincidence	Sheet cavitation (in separation zone) and vortex cavitation (within CGUs)	[155]
Reactor optimization (CFD)	*c*: 1–1.5 mm, *ω*: 12°, *N*: 2, *γ*: 2°, *r*: 13 mm	Highest cavitation efficiency	Optimized CGU layout enhances bubble collapse	[156]
Cavitation-vortex (CFD)	–	Complex spiral and vortex flows trigger sheet and vortex cavitation	Vorticity transport dominated by stretching/expansion	[141]
Wastewater (*E. coli*, *E. faecalis*)	0–3000 rpm; ≤10 min	Complete bacterial inactivation	Cavitation jets/shock rupture cells; ^•^OH oxidizes	[140]
Sludge + pig slurry	5600 rpm; 100 L/min; No cooling (60–70 °C); 240 min	Disintegration: 17.4 %; SCOD:3500 mg/L increase; volatile fatty acids (VFAs) significantly enhanced.	Collapse heat/pressure disrupt cell walls, release organics	[157]
Industrial wastewater	2500 rpm; lead angle: 75°/8°; gap: 2 mm	bubble: 54 %increase (75°), enhancing cavitation cloud formation and suppressing short-circuit flow	Rotor–stator serrations enhance collapse and flow control	[158]
Textile wastewater	8 serration rows (15 each); 180 mm outer diameter; 105° serration face angle	Maximized cavitation performance	Structural design intensifies cavitation	[136]
Tap water	Grooves: 6 * 3 mm; serrations: 18°; 3800 rpm	Enhanced cavitation intensity	Attached + shear cavitation synergize	[159]

## 7 Other process intensification

Cavitation, beyond its established applications in biofuel production, droplet emulsification, food processing, and wastewater sludge treatment, has also been shown to significantly enhance mass and heat transfer efficiencies [160], making it suitable for process intensification in reactions [97,161]. Moreover, cavitation can modulate material structures at the molecular level, thereby improving production capacity and product quality [162,163]. As such, cavitation is not merely a physical treatment technique but also a multidimensional, multifunctional green intensification technology with broad application prospects and strong potential for future development.

Kwon and Yoon [67] measured heat generation of a serrated-disk rotational cavitator at 2100–3600 rpm and 0.99–2.16 bar. Cavitation required inlet pressure >1.25 bar and speed >2700 rpm; raising either variable increased heat output, with speed more influential. Peak thermal efficiency reached 90.42 %, far above electric heating, and lower cavitation number correlated with higher heat. Without an electric motor, the unit can couple directly to hydro/wind turbines. Applying hydrodynamic cavitation to pulping, Badve et al. [164] combined an indentation-type cavitator with alkali pretreatment for wheat-straw delignification: 10–15 min raised paper tensile index by 50–55 %, and higher speed enhanced delignification and strength. To clarify mechanisms, Badve et al. [70] used CFD and experiments with the Weissler reaction (KI decomposition). Higher speed increased shear rate but narrowed high-shear zones and stabilized flow; cavitation was strongest at 2200–2500 rpm, releasing up to 50 ppm iodine. (γ is the shear rate: $\gamma =\sqrt{\frac{P}{\mu V}}$, where *P* is the input power, *V* is the fluid volume, and *μ* is the dynamic viscosity. The shear rate characterizes the velocity gradient between the rotor and stator and reflects the intensity of the local flow field.) For geometry effects in the indentation-type cavitator, Zhang et al. [165] showed that indentation diameter governs vapor–liquid exchange and, when increased, markedly amplifies cavitation intensity and fluctuations; indentation height controls cavitation area/intensity, while conical-base length sets vortex number and strength. Complementarily, Wang et al. [166] found that larger indentation diameter expands low-pressure regions, strengthens vortices, intensifies phase change, and thus promotes cavitation.

**Fig. 25:**
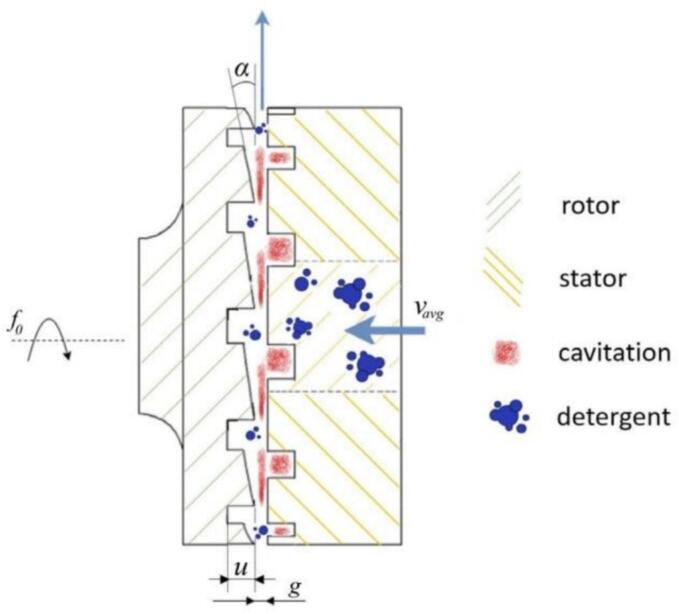
Schematic diagram illustrating the function of a radial serrated rotational cavitator [167].

Stepišnik Perdih et al. [167], building on their earlier work [145], used a radial-serrated rotational cavitator to accelerate detergent dissolution in washing machines (Fig. 25). Cavitation markedly increased the dissolution rate, reaching >80 % within 10 s; mechanical action was the main driver. Integrating the device into washers could shorten cycles and enable more sustainable laundering. Similarly, Kosel et al. [168] applied the serrated cavitator to industrial 3 % softwood-fiber pulp beating. Unlike orifice/Venturi units, which suffer high pressure losses and clogging, the cavitator improved drainage and paper strength, achieving a tensile index of 50.5 kN m/kg and a burst index of 3 kPa m^2^/g. Sun et al. [68] analyzed flow and cavitation over a hexahedral-textured rotating disk and showed that texture-induced local low pressure promotes cavitation and lubrication, whereas deeper dimples reduce shear stress and suppress cavitation—guiding surface optimization for disk-type hydraulic machinery. Extending to gas–liquid transfer, Giuliano et al. [169] employed rotational cavitation for bio-hydrogenotrophic methanation (BHM) of H_2_ and CO_2_ with methanogenic archaea, achieving near-100 % H_2_ utilization, >99 % CH_4_ purity, and stable performance over 160 days, highlighting strong advantages for mass-transfer enhancement and long-term feasibility (see Table 10 for further details).

**Table 10:** Applications of rotational cavitators in other process intensification.

Material	Parameters	Performance	Mechanism	Ref.
Water	3300 rpm; inlet pressure: 2.04 bar	Thermal efficiency: 90.42 %	Bubble collapse generates localized heat; mechanical energy converted into thermal	[67]
Wheat straw (KOH-pretreated)	2200–2700 rpm; 15 min; slurry consistency: 1–7 %	Tensile index: 45–66 % increase; cardboard grade met; energy: 50 % reduction vs. conventional	Shear/turbulence disrupt lignin; ^•^OH radicals promote hydrolysis and hydrophilicity	[164]
KI solution	2200–2500 rpm	Iodine release: 50 ppm	^•^OH radicals from bubble collapse decompose KI; indicator of cavitation intensity	[70]
Water (simulation & experiment)	Indentation diameter: 17 mm; height: 55 mm; bottom length: 1 mm	Pressure fluctuation dominant at 24*f*_i_; max amplitude near indentation top	Indentation geometry alters vortex/pressure: diameter–vapor exchange, height–cavitation zone, cone–vortex number/strength	[165]
Water (CFD)	1200 rpm; indentation diameter: 11–19 mm	At *d* = 19 mm, cavitation region expanded, more vortices, stronger turbulence	Larger indentation expands low-pressure zones, enhances vapor–liquid exchange and turbulence	[166]
Detergent solution	7000 rpm	80 % dissolved in 10 s (vs. 150 s without cavitation)	Bubble collapse produces microjets/pulses; accelerates solute–solvent interaction and diffusion	[167]
Softwood fiber slurry	6000 rpm; 20 min; consistency: 3 %	Improved dewatering; tensile index: 50.5 kN·m/kg; burst strength: 3 kPa·m^2^/g	Shear/cavitation break H-bonds, promote fibrillation; serrations create multi-zone cavitation clouds	[168]
Hydraulic oil	depth: 0.05 mm; 6000 rpm	Improved lubrication and dynamic pressure	Shear cavitation at textured boundaries/low-pressure vortices increases dynamic pressure	[68]
H_2_/CO_2_ (80:20 v/v)	3528 rpm	Nearly 100 % H_2_ utilization; CH_4_ >99 %; no recycling needed	Cavitation microbubbles enlarge gas–liquid area, enhance H_2_/CO_2_ dissolution	[169]

## 8 Insights and perspectives on rotational cavitator systems

After gaining an in-depth understanding of the various applications of rotational cavitation, it is necessary to systematically analyze this technology from the perspectives of cavitation induction mechanisms and energy consumption. On the one hand, the cavitation induction mechanisms of different types of rotational cavitators determine the internal flow patterns and cavitation intensity, and thus are key to explaining performance differences between devices. On the other hand, the energy-saving effects associated with cavitation during process intensification have an important impact on the economic viability and sustainability of engineering applications. In addition, discussing the limitations of rotational cavitators helps to more objectively assess the actual benefits and applicability of this technology. Therefore, this section focuses on cavitation mechanisms, energy consumption characteristics of different devices, and the main operational limitations, with the aim of providing a comprehensive evaluation of the operating characteristics and application value of rotational cavitators and offering guidance for device selection in different application fields.

### 8.1 Cavitation mechanisms

To clarify the cavitation-inducing mechanisms of different rotational cavitators, the key principles are summarized below to support deeper insight into their operating features and to guide equipment selection across applications (see Table 11 for further details). The indentation-type cavitator is the most widely used due to its simple rotor design with internal indentations. It has been applied in biofuel production, droplet emulsification, food processing, and sludge treatment. Cavitation arises when liquid passes through the rotor–stator gap at high velocity, enters the rotor indentations, and undergoes sudden channel contraction, which sharply lowers local pressure. Centrifugal outflow from the indentations further generates low-pressure regions near the indentation edges, initiating cavitation; indentation size, height, and arrangement strongly affect intensity. The radial-serrated cavitator, typically with rotor–stator or dual-rotor configurations, is used in sludge treatment and process intensification. Here, aligned serrations form a Venturi-like throat, forcing liquid into radial and tangential motion. Radial flow ensures suction and continuity, while tangential flow drives rotation in the chamber. The narrow gaps and sharp edges impose high shear stress and periodic pressure pulsations, producing shear-induced cavitation. In contrast, the axial-serrated cavitator is a newer design applied in sludge processing and emulsification. Multiple rotor–stator pairs distributed axially enhance periodic shear. Fluid accelerated into serrated grooves collides with walls and interacts with injected streams, forming vortices and low-pressure zones that trigger cavitation. This layout suppresses stagnation and short-circuit flow while promoting vigorous bubble collapse.

**Table 11:** Cavitation-inducing mechanisms of various rotational cavitators.

cavitator type	Cavitation mechanism	Application fields
Indentation-type rotational cavitator	Fluid accelerates through the rotor indentation, causing a sharp pressure drop; centrifugal force drives the fluid out, creating low-pressure regions and inducing cavitation	Biofuel production, droplet emulsification, food processing, wastewater sludge treatment, and other process intensification fields
Radial serrated rotational cavitator	High-speed rotation of rotor serrations induces radial and tangential motion; narrow gaps and sharp edges generate high shear stress and periodic pressure fluctuations, triggering shear cavitation	Wastewater sludge treatment, process intensification
Axial serrated rotational cavitator	Multiple pairs of axially distributed rotor–stator structures enhance fluid shear; fluid in the serration grooves impacts walls and interacts with injected flow, forming vortices and low-pressure zones to induce cavitation	Wastewater sludge treatment, droplet emulsification
Pinned-disk rotational cavitator	Staggered cylindrical pins cause fluid separation and pulsating gap flow; downstream vortex shedding generates abrupt pressure fluctuations, enhancing cavitation	Wastewater treatment
Hybrid-geometry rotational cavitator	Cavitation is jointly induced by indentations and shear-interlaced structures, exhibiting flow characteristics distinct from the previously described devices	Wastewater treatment, food disinfection, droplet emulsification

Unlike serrated cavitators, which induce cavitation via Venturi-like constrictions created by the periodic interlocking of serrations, the pinned-disk reactor relies on cylindrical pins arranged on the rotor–stator disks. The staggered pin array causes flow separation and a pulsating gap flow; vortices shed downstream of each pin. The resulting downstream pressure pulsations intensify cavitation. This design is used mainly in wastewater treatment. Emerging hybrid-geometry cavitators combine indentation- and shear-induced mechanisms and show distinct flow features. In the cavitator designed by Sun et al. [153], periodic interaction between indentations on the rotor and stator drives radial flow into moving and stationary cavities, forms vortices that strike cavity trailing edges, and generates pressure disturbances that trigger cavitation; sheet and vortex cavitation appear cyclically. Similarly, the device by Song et al. [107], with through-grooves on both rotor and stator, induces cavitation through the joint action of shear separation and local low pressure. Beyond research prototypes, commercial rotational cavitators are increasingly adopted as mainstream experimental tools.

### 8.2 Device energy consumption

To enable a meaningful comparison of different types of cavitators across application fields, their respective energy consumption must be further considered and summarized in Table 12. This aims to highlight their energy-saving advantages and high-efficiency characteristics, thereby providing a more comprehensive view of the practical value and potential of this technology.

**Table 12:** Energy consumption of different types of rotational cavitators in applications.

Application	Type of rotational cavitator	Cavitator energy consumption	Conventional method energy consumption	Ref.
Biodiesel	Indentation-type	12.5 W·h/kg	183 W·h/kg (orifice hydrodynamic); 250 W·h/kg (ultrasonication); 500 W·h/kg (mechanical stirring)	[81]
		0.0264 kWh/L (low-cost 3D-printed rotor)	/	[64]
		0.137 kWh/L, ∼3.71 $/h methanol cost saving	/	[89]
		Methanol and sulfuric acid costs:0.94 $/h and 0.10 $/h reduction	/	[56]
	Radial serrated	0.030 kWh/L	0.222 kWh/L	[78]
	SME SC Progen IMPEX SRL	Specific energy: 629 J/mL(50 s)	/	[74]
Biogas	Indentation-type	470 kJ/kg TS (full-scale test)	/	[97]
		271.21 kJ/kg TS (full-scale test)	/	[98]
	Radial serrated	∼0.0003 kWh/L (calculated from data)	/	[83]
Droplet emulsification	Indentation-type	3D-printed rotor: low cost (∼2 USD)	Stainless steel: ∼600 USD each	[25]
	Hybrid-geometry	Energy: ∼25 % of ultrasonication; effective power: 13.20 kW; energy: 6.60 kWh	/	[109]
	E-PIC S.r.L. (Mongrando, Italy)	4.6 kWh/kg	Conventional heating: 15 kWh/kg; laboratory device: 7.4 kWh/kg	[110]
Food processing	Indentation-type	<220 kJ/kg; maximum efficiency: 84 %	/	[121]
	Hybrid-geometry	∼214.29 kJ/L(calculated from data); production cost: 0.00268 USD/L	/	[116]
	APV CavitatorSPX flow technology, Silkeborg, Denmark	∼170 kJ/L	/	[122]
Wastewater sludge	Indentation-type	Cost-effectiveness and time efficiency: 100 times higher	/	[143]
		Energy consumption: 0.56 kWh/m^3^; Rs.2.8/m^3^	/	[135]
		Cost: Rs. 7.28 (with O_3_)	/	[144]
	Radial serrated	0.01–0.04 kWh/m^3^/order	0.99–6.94 kWh/m^3^/order	[147]
		67.2 kWh/m^3^/order (Bacillus subtilis)	Single-serration rotor: 347.5 kWh/m^3^/order	[148]
		1.9 kg COD treatment cost: 1 EUR	/	[149]
	Axial serrated	3.58 × 10^4^–2.85 × 10^3^ kJ/kg TS	Orifice plate: 1.22 × 10^5^ kJ/kg TS; ultrasonication: 1.83 × 10^6^ kJ/kg TS	[157]
	Pinned-disk	48.3 kWh/kg COD	30 passes: 24 % higher energy per kg COD, 4.1× (310 %) lower COD removal capacity	[138]
		8.2 kWh/kg COD	/	[139]
		1.41 × 10^3^ ± 0.16 × 10^3^ kJ/kg TS; 89 ± 4 kJ/g sCOD	/	[152]
	Hybrid-geometry	41 J/mL; energy efficiency: 43 %	Ultrasonication: 126 J/mL; high-pressure homogenization: 1023 J/mL	[142]
		Thermal efficiency: 82.18 %; cost: $3.019/m^3^	/	[153]
		Specific energy: 84,530–329,800 kJ/kg TS (5–20 passes)	/	[19]
		Thermal efficiency: 79.99 %; treatment cost: $2.72/m^3^	140 × lower treatment rate; 50 × higher cost	[66]
		0.748 kWh; 0.0499 kWh/L;	/	[57]
		0.06–0.12 kWh/L	Ultrasonication: 0.124–1.61 kWh/L; orifice plate: 1.49 kWh/L	[140]
Other process intensification	Indentation-type	1785 kWh/m^3^/order	Kraft pulping: 3942 kWh/m^3^/order; steam explosion: 3482 kWh/m^3^/order	[164]
	Radial serrated	41.4–58.4 kWh/m^3^/order	87.1 kWh/m^3^/order (Valley pulper)	[168]
	Hybrid-geometry	1.213 × 10^4^–5.271 × 10^4^ kJ/h	/	[67]

### 8.3 Practical limitations and challenges

Different types of rotational cavitators exhibit distinct advantages across various application scenarios and can be selected according to specific process requirements. In early developments, indentation-type rotational cavitators were widely adopted due to their simple geometry and strong operational adaptability, becoming the mainstream choice in commercial systems. As the demand for intensified cavitation increased, serrated configurations gradually emerged in high-intensity applications such as wastewater treatment and sludge disintegration. Building upon this concept, pinned-disk designs were subsequently introduced and demonstrated superior cavitation performance. In recent years, hybrid-geometry rotational cavitators have shown even stronger cavitation intensity and material processing capability, thereby promoting greater diversification in reactor structural design.

However, it should be noted that despite the strong performance of rotational cavitators in process intensification and energy enhancement, their engineering-scale application still faces several limitations. First, different material systems exhibit markedly different sensitivities to cavitation intensity, shear environment, and pressure fluctuations, and optimal operating conditions often depend on multi-parameter coupling (e.g., rotational speed, geometric configuration, temperature, and residence time). In some cases, co-application with oxidants is still required to achieve higher energy efficiency than cavitation alone. Second, compared with structurally simple non-rotational cavitation devices such as orifice plates and Venturi tubes, rotational cavitators consist of rotor–stator assemblies with higher structural complexity, making cost, maintenance, and operational reliability critical considerations. Although the high shear environment generated by high-speed rotation facilitates process intensification, it may also damage fragile structures or sensitive biological systems.

Moreover, the repeated generation and collapse of cavitation bubbles can induce material fatigue and localized erosion during long-term operation, leading to wear of the rotor, stator, and gap regions. Such degradation may compromise device stability and reduce the reproducibility of cavitation intensity. For solid-containing systems, high solids content may cause gap blockage or accelerate abrasive wear, while also disturbing the internal flow field. In practical applications, the potential for further improvement in overall energy efficiency remains to be fully explored.

## 9 Concluding remarks

Rotational cavitation is increasingly recognized as a versatile and effective process-intensification technology, offering controllable cavitation dynamics, reduced energy requirements, and broad applicability in environmental remediation, food processing, and biochemical operations. In comparison with conventional hydrodynamic and ultrasonic systems, rotational cavitators deliver higher cavitation intensity, improved scalability, and superior overall process efficiency. Advances in device geometries—such as indentation structures, serrated profiles, and pinned-disk configurations—allow sustained cavitation intensity under mild, continuous, and scalable operating conditions, thereby enhancing key processes including microbial inactivation, emulsification, and component release. Furthermore, coupling rotational cavitation with chemical oxidants further expands its utility in strengthening reaction processes, offering significant value for advanced and sustainable process intensification.

Research on rotational cavitators is shifting from macroscopic observation to microscopic mechanism. Significant advances have been made in cavitation induction, pattern evolution, and action mechanisms. In recent years, the combined use of experiments and numerical simulation has improved visualization and system-level analysis. This synergy deepens understanding of cavitation onset and development and provides theoretical support for mechanism elucidation. These insights lay a solid foundation for green applications of rotational cavitation in efficient mixing, mass-transfer enhancement, process intensification, and pollution control.

Collapse of cavitation bubbles generates extreme temperature, pressure, shear, and turbulence. These conditions intensify mixing, mass transfer, and reaction kinetics, accelerating transesterification, hydrolysis, and anaerobic digestion, and increasing biofuel conversion and yields. In emulsification and liquid-food processing, high shear and turbulence promote droplet breakup and size reduction, improving efficiency and stability. Cavitation also drives microbial inactivation, structural disruption, and component release, which improves rheology and enhances nutritional and functional qualities while raising processing efficiency. In water and environmental treatment, bubble collapse and radical formation enable effective disinfection, particle disintegration, and oxidative degradation of pollutants, markedly increasing purification efficiency. In addition, cavitation enhances heat and mass transfer across many reactions and can modulate structure at the molecular scale, supporting higher throughput and better product quality.

In summary, rotational cavitators, as an important emerging direction in cavitation technology, show substantial potential in mechanistic understanding, structural design, and practical applications. Different rotor types and channel geometries exert significant influence on cavitation behavior. Future development of rotational cavitators will require continued advancement across several key areas. First, the understanding of flow-field mechanisms and cavitation evolution remains limited; thus, high-resolution experimental diagnostics and CFD simulations are needed to further elucidate vortex structures, cavitation cloud dynamics, and unsteady coupling mechanisms. Second, structural optimization, material selection, and economic feasibility remain major bottlenecks restricting long-term stable operation. It will be necessary to develop more rational geometric designs and cost-effective, corrosion-resistant material systems that ensure cavitation performance while achieving an improved balance among efficiency, durability, and manufacturing or maintenance costs. Furthermore, multi-field coupling (e.g., ultrasound) offers additional opportunities to enhance cavitation intensity and reaction selectivity.

Current industrial processes remain constrained by the low energy efficiency and limited intensification capacity of conventional reactors, underscoring the growing need for rotational cavitation technologies. Although rotational cavitators have demonstrated promising energy performance and application potential at laboratory and pilot scales, their widespread industrial implementation still requires substantive advances in energy optimization, scale-up design, material durability, and overall economic viability. Achieving these breakthroughs will be essential for improving cross-sector applicability and enabling efficient, stable, and scalable deployment of rotational cavitation technologies.

## CRediT authorship contribution statement

**Yu-Hang Zhang:** Writing – original draft, Methodology, Investigation, Formal analysis, Data curation. **Zhi-Ying Zheng:** Writing – review & editing, Supervision, Project administration, Methodology, Funding acquisition, Conceptualization. **David Ezekoye:** Investigation, Formal analysis, Data curation. **Lu Wang:** Writing – review & editing, Project administration, Methodology, Conceptualization. **Li-Ming Yao:** Resources, Conceptualization. **Vladimir A. Kulagin:** Supervision, Resources, Conceptualization. **Jian Wu:** Supervision, Resources.

## Declaration of competing interest

The authors declare that they have no known competing financial interests or personal relationships that could have appeared to influence the work reported in this paper.
